# Genetic architecture of photosynthesis in *Sorghum bicolor* under non-stress and cold stress conditions

**DOI:** 10.1093/jxb/erx276

**Published:** 2017-08-18

**Authors:** Diego Ortiz, Jieyun Hu, Maria G Salas Fernandez

**Affiliations:** Department of Agronomy, Iowa State University, Ames, IA, USA

**Keywords:** Chlorophyll fluorescence, cold, genome-wide association study, photoprotection, photosynthesis, sorghum

## Abstract

Sorghum (*Sorghum bicolor* L. Moench) is a C_4_ species sensitive to the cold spring conditions that occur at northern latitudes, especially when coupled with excessive light, and that greatly affect the photosynthetic rate. The objective of this study was to discover genes/genomic regions that control the capacity to cope with excessive energy under low temperature conditions during the vegetative growth period. A genome-wide association study (GWAS) was conducted for seven photosynthetic gas exchange and chlorophyll fluorescence traits under three consecutive temperature treatments: control (28 °C/24 °C), cold (15 °C/15 °C), and recovery (28 °C/24 °C). Cold stress significantly reduced the rate of photosynthetic CO_2_ uptake of sorghum plants, and a total of 143 unique genomic regions were discovered associated with at least one trait in a particular treatment or with derived variables. Ten regions on chromosomes 3, 4, 6, 7, and 8 that harbor multiple significant markers in linkage disequilibrium (LD) were consistently identified in gas exchange and chlorophyll fluorescence traits. Several candidate genes within those intervals have predicted functions related to carotenoids, phytohormones, thioredoxin, components of PSI, and antioxidants. These regions represent the most promising results for future validation and with potential application for the improvement of crop productivity under cold stress.

## Teaser

Multiple genomic regions explain variation in the carbon fixation response to non-stress and cold stress in *Sorghum bicolor*, with some of these regions simultaneously controlling multiple traits.

## Introduction

Sorghum (*Sorghum bicolor* L. Moench) is a crop of tropical origin that has been successfully introduced to high latitudes. However, spring conditions can expose early stages to cold events that negatively affect germination and growth, reducing sorghum biomass production and yield (Franks *et al.*, 2006; Maulana and Tesso, 2013). In order to expand the growth period and increase productivity, it is essential to develop sorghum cultivars better adapted to cold conditions.

Decreases in growth can be explained by the sensitivity of the photosynthetic apparatus of C_4_ species such as sorghum and maize (*Zea mays*) to low temperatures (Taylor and Rowley, 1971; Long *et al.*, 1983; Wang *et al.*, 2008; Fiedler *et al.*, 2014). Temperatures below 20 °C cause chilling stress in sorghum, which greatly affect the agronomic performance of this crop (Peacock, 1982). Rubisco activity is a major limitation to carbon assimilation in temperatures below 20 °C due to a reduced Rubisco capacity and content in C_4_ compared with C_3_ plants (Sage, 2002; Kubien *et al.*, 2003). Reductions in leaf photosynthetic rate are also related to instability of pyruvate phosphate dikinase (PPDK) under cold conditions, which impairs the capacity to regenerate phosphoenolpyruvate in mesophyll cells (Naidu *et al.*, 2003; Naidu and Long, 2004; Wang *et al.*, 2008). However, there are multiple C_4_ grasses adapted to cold conditions, such as miscanthus (*Miscanthus×giganteus*) and *Spartina cynosuroides*, for which an increase in Rubisco and PPDK content has been reported when exposed to low temperatures (Naidu *et al.*, 2003; Wang *et al.*, 2008).

A cold condition, coupled with excessive light, can lead to photoinhibition (Haldimann *et al.*, 1996; Pimentel *et al.*, 2005) and drive the transfer of energy from chlorophyll to oxygen, creating harmful reactive oxygen species. However, plants have developed several mechanisms to protect themselves from excessive light, generally called photoprotection. Plants can reduce light absorption through chloroplast movement (Kagawa and Wada, 2002) and reduction of antenna size (Bailey *et al.*, 2001). Additionally, carotenoid pigments can dissipate the excess energy through the xanthophyll cycle, in which violaxanthin is converted into zeaxanthin. This generates a conformational change in the antenna of PSII that allows the plant to dissipate excess energy as heat (Demmig-Adams *et al.*, 1989). Other important photoprotection processes include the cyclic electron flow around PSI (CEF) that prevents the over-reduction in the electron transport pathway (Munekage *et al.*, 2004), and the repair of damaged D1 protein of PSII by the ATP-dependent metalloprotease FtsH (Lindahl *et al.*, 2000). Although mutant studies in model species have revealed important genes affecting these processes, their natural allelic variation in commercial crops remains unexplored (Long *et al.*, 2006; Zhu *et al.*, 2010; Takahashi and Badger, 2011).

Most of the photoprotection mechanisms affect efficiencies of the light reactions, which can be assessed through chlorophyll fluorescence quenching analysis. The use of portable pulse amplitude-modulated chlorophyll fluorometers enables a deeper understanding of the functioning of the photosynthetic apparatus. Coupled with gas exchange measurements, chlorophyll fluorescence has facilitated the characterization of the *in vivo* response of photosynthesis to a variety of stress conditions, including drought (Kiani *et al.*, 2008; Gu *et al.*, 2012*a*), salinity (Netondo *et al.*, 2004), and cold (Nie *et al.*, 1992; Fracheboud *et al.*, 1999; Kościelniak *et al.*, 2005; Savitch *et al.*, 2009; Strigens *et al.*, 2013) in several crops.

There is evidence of important photosynthesis and chlorophyll fluorescence variability in sorghum (Kidambi *et al.*, 1990; Peng and Krieg, 1992; Henderson *et al.*, 1998; Balota *et al.*, 2008; Fiedler *et al.*, 2014; Salas Fernandez *et al.*, 2015), suggesting opportunities for selection and genetic improvement. Several studies have reported quantitative trait loci (QTLs) for germination and early growth parameters under cold conditions (Knoll *et al.*, 2008; Burow *et al.*, 2010; Fiedler *et al.*, 2012; Bekele *et al.*, 2014), but photosynthesis and photoprotection were not evaluated. Due to the complex nature of these physiological processes, quantitative genetic studies are limited but essential if improvement in photosynthetic capacity under stress is ever going to become a breeding goal. Even though selection for cold tolerance using chlorophyll fluorescence has been successful in maize (Fracheboud *et al.*, 1999; Kościelniak *et al.*, 2005), there is a significant knowledge gap about the genetic structure that controls carbon assimilation and photoprotection parameters under non-stress or cold conditions in sorghum. The objective of this work was to utilize natural variation within the species to discover genomic regions or genes that regulate the ability of sorghum plants to cope with excessive energy under low temperature stress conditions during the vegetative growth period. Closing this knowledge gap about the genetic control of photoprotective capacity could significantly boost the improvement of crop productivity under cold stress, particularly important and frequently present early in the growing season at northern latitudes. Additionally, the knowledge generated in this study could be leveraged by comparative genomics between sorghum and other grass species such as rice, maize, sugarcane, and wheat.

## Materials and methods

### Germplasm

A total of 304 accessions from the Sorghum Association Panel were used in this study (Casa *et al.*, 2008). This panel comprises accessions from major cultivated races and represents the genetic diversity of the crop. This panel has been used in association studies for traits such as plant architecture (Morris *et al.*, 2013; Mantilla Perez *et al.*, 2014; Zhao *et al.*, 2016), and grain quality (Sukumaran *et al.*, 2012).

### Experimental design

The panel was arranged in an incomplete block design with eight sets or incomplete blocks of 39 accessions each. Sets were grown consecutively, and two growth chambers were used as replicates. Four accessions (PIs: 534021, 597966, 655975, and 548797) were included as checks in all sets to correct for set and chamber effects. They were selected based upon morphological differences (i.e. leaf size and shape) and photosynthetic response to cold from preliminary assays (data not shown). Accessions were randomized within each growth chamber and they were also randomized relative to the time of measurement.

### Growth conditions

Two plants per accession were grown in 6 liter pots in a greenhouse. The photoperiod was set to 16 h using supplemental light, and air temperatures were set to 28 °C/ 24 °C for day and night, respectively. Plants were grown in Metro Mix 900 soil (Sun Gro Horticulture) and fertilized with Peters Excel Cal-Mag Fertilizer (15-5-15) every 1 d or 2 d as needed. Miracid fertilizer (Miracle Gro^®^) was applied every 2 weeks to correct for high pH in the water source, and a solution of 220 ppm Ca^2+^/96 ppm Mg was applied once to prevent Ca^2+^ deficiencies.

Fifteen days after planting, the two plants per accession were transferred to each of two growth chambers (Percival, model PGW36T, capacity 11.28 m^3^) equipped with both high pressure sodium and metal halide lamps. Growth chambers, used as replicates, were set to identical conditions of 50% air relative humidity, 28 °C day /24 °C night temperatures, and a photoperiod of 16 h, with light levels increasing from 07.00 h to 08.00 h and decreasing from 17.00 h to 22.00 h, to mimic sunrise and sunset conditions. Additionally, when introduced from the greenhouse, plants were gradually adapted for 2 weeks from an initial photosynthetically active radiation (PAR) of 400 μmol photons m^–2^ s^–1^ to a final value of 1000 μmol photons m^–2^ s^–1^.

Data were collected from 30-day-old plants during three temperature treatment periods: control or non-stress (28 °C/24 °C for 6 d), cold stress (15 °C/15 °C for 7 d), and recovery (28 °C/24 °C for 5 d). For details, see Supplementary Fig. S1 at *JXB* online.

### Photosynthesis and fluorescence

Gas exchange and fluorescence parameters were collected using three LI-COR 6400XT portable open-path gas analyzers equipped with a 6400-40 Leaf Chamber Fluorometer (LI-COR, Lincoln, NE, USA). Data were obtained at the following time points: (i) control period: day 1, 3, and 6; (ii) cold stress period: day 3 and 7; and (iii) recovery period: day 3 and 5.

Fluorescence parameters were measured in the uppermost fully expanded leaf that was dark adapted by covering with aluminum foil the night before. Measurements were taken in a younger expanded leaf in the cold and recovery treatments in order to prevent any aging effect. Minimum chlorophyll fluorescence (*F*_0_) was determined using a modulating radiation of three, and maximum chlorophyll fluorescence (*F*_m_) was obtained with a saturating pulse of 8000 μmol photons m^–2^ s^–1^ for 0.8 s in the dark-adapted state. Variable chlorophyll fluorescence (*F*_v_) was calculated as *F*_m_–*F*_0_, and maximum quantum yield of PSII as *F*_v_/*F*_m_.

The same leaf was used for light- and dark-adapted measurements on the same day. Overnight dark-adapted fluorescence was measured at the start of the day (06.30 h to 08.00 h). After at least 30 min of light, gas exchange and fluorescence parameters were measured between 09.00 h and 14.00 h. Conditions in the LI-COR 6400XT leaf chamber cuvette were set to 1000 μmol photons m^–2^ s^–1^ PAR, 400 ppm reference CO_2_ concentration, and 50–60% relative humidity. Leaf temperature was set to 28 °C during control and recovery days, and 15 °C during cold days. The fraction of blue light was 10% of the PAR level to maximize stomatal aperture. Light-adapted minimum chlorophyll fluorescence (*F*_0_') and maximum chlorophyll fluorescence (*F*_m_') were determined using a measuring light of three and a saturating pulse of 8000 μmol photons m^–2^ s^–1^ for 0.8 s, respectively. Four stability parameters were monitored for each measurement [leaf carbon assimilation rate (*A*), stomatal conductance (*g*_s_), steady-state fluorescence, and water vapor concentration] and data were recorded when they reached stability, namely after a minimum of 3 min and when the coefficient of variation was <1.2. The following gas exchange parameters were recorded: *A*, *g*_s_, and transpiration rate (*E*). Fluorescence parameters included: effective quantum yield of PSII (Φ_PSII_), efficiency of energy captured by open PSII reaction centers (*F*_v_'/*F*_m_'), and photochemical quenching or fraction of PSII reaction centers that are open (*qP*) (Genty *et al.*, 1989; Maxwell and Johnson, 2000).

Finally, for each of the previously described parameters, we defined the following derived variables: (i) ‘cumulative response’; (ii) the ratio between control and cold stress values; and (iii) the ratio between cold and recovery performance. The ‘cumulative response’ integrates the physiological performance of lines over all time periods and was estimated as the area under the seven-point measurement curve (graphically defined in Supplementary Fig. S2). Considering that different accessions have variable photosynthetic capacity under non-stress, this derived variable facilitates the evaluation of the stress response relative to the non-stress values.

### Statistical analysis

Variables were analyzed by treatment using the following number of replications: six for the control period (two plants and three time replications per plant), four for the cold treatment (two plants and two time replications per plant), and four for the recovery period (two plants and two time replications per plant). Variables were also analyzed over all treatments (called ‘cumulative response’) using a total of 14 data points per genotype (two plants, seven measurements over treatments), and as a ratio between control and cold and between cold and recovery treatments. Statistical analyses were performed using SAS version 9.4 (SAS Institute, Cary, NC, USA). Days of measurement were considered a replication over time within each temperature treatment period. Best linear unbiased predictions (BLUPs) were estimated for each accession and physiological parameter using Proc MIXED. The model for *A*, *E*, *g*_s_, *qP*, Φ_PSII_, and *F*_v_'/*F*_m_' for each evaluation period (control, cold, and recovery) was:

$${\text{Y}}_{\text{ijklm}}=\mu +{\text{S}}_{i}+{\text{R}}_{\left(\text{i}\right)\text{j}}+{\text{D}}_{k}+{\text{G}}_{l}+{\text{M}}_{m}+\varepsilon_{\text{n}}$$ (1)

where Y_ijklm_ is the response variable, μ is the overall mean, S_i_ is the set effect, R_(i)j_ is the replication nested in set effect (that corresponds to growth chamber effects), D_k_ is the day effect for each period, G_l_ is the genotype (accession) effect, M is the machine (gas analyzer) effect, and ε_n_ is the residual.

For *F*_v_/*F*_m_, the machine effect was not significant, and thus it was excluded from the final model. The *F*_v_/*F*_m_ model for each of the three treatment periods was:

$${\text{Y}}_{\text{ijklm}}=\mu +{\text{S}}_{i}+{\text{R}}_{\left(\text{i}\right)\text{j}}+{\text{D}}_{k}+{\text{G}}_{l}+\varepsilon_{\text{m}}$$ (2)

Finally, there was a small but significant effect of leaf temperature on *A* during the cold period, so it was included as a covariate in the model for these two variables. In the different models described in Equations 1 and 2, day and machine were considered fixed effects while set, replication nested in set, and genotype effects were treated as random.

In all cases, BLUPs obtained as described above were further used as phenotypic values in a genome-wide association study (GWAS). Descriptive statistics and correlations coefficients were estimated using PROC CORR in SAS.

Heritability (*h*^2^) was estimated for each trait as:

$$h^{\text{2}}=\sigma^{\text{2}}{}_{\text{G}}/[\sigma^{\text{2}}{}_{\text{G}}+(\sigma^{\text{2}}{}_{\varepsilon }/\text{r})]$$

where σ^2^_G_ is the genotypic variance, σ^2^_ε_ is the error variance, and r is the number of replications. Heritability, as estimated herein, provides a quantification of data repeatability.

### GWAS

A mixed linear model was fitted for each variable using TASSEL software version 5.2.12. This model accounts for population structure (Q, fixed) and kinship (K, random) effects to minimize spurious associations (Zhang *et al.*, 2010). Q and K were calculated using STRUCTURE 2.2.3 (Pritchard *et al.*, 2000) and SPAGeDi 1.4 (Hardy and Vekemans, 2002), respectively, as described in Mantilla Perez *et al.* (2014).

The original public data set of ~260000 single nucleotide polymorphisms (SNPs) (http://www.morrislab.org/data) generated using genotyping-by-sequencing (GBS) (Elshire *et al.*, 2011; Morris *et al.*, 2013) was filtered based on two criteria: missing data <40% and minor allele frequency >5%. A final set of 134600 SNPs was subsequently used in a GWAS for all traits. Additionally, markers for photosynthesis and photoprotection *a priori* candidate genes were investigated according to Mantilla Perez *et al.* (2014). In summary, genes were first identified in model species, and protein sequences were obtained from the National Center for Biotechnology Information databases. TBLASTN was used to search homologous sequences in the sorghum genome from phytozome V1.4 (Paterson *et al.*, 2009). If no GBS markers were available for the targeted candidate genes, new SNPs were developed and genotyped using an SNPtype™ Assay (Fluidigm) on the Fluidigm BioMark™ system and GT 96.96 Dynamic Array Integrated Fluidic Circuits of Fluidigm. A total of 188 markers (considering both GBS and Fluidigm data sets) within or 5 kb upstream/downstream of 67 photosynthesis and photoprotection candidate genes were included in the GWAS. A detailed description of genes and markers is provided in Supplementary Table S11. Additionally, we explored genes within 55 kb from significant SNPs, considering that, in sorghum, the linkage disequilibrium (LD) decay to background levels (*r*^2^<0.1) has been reported to be within 15–150 kb (Hamblin *et al.*, 2005; Bouchet *et al.*, 2012; Morris *et al.*, 2013).

The trait-specific significance threshold was established based on the false discovery rate, calculated using QVALUE software (Storey and Tibshirani, 2003), to control for false-positive associations due to multiple comparisons.

### RT–PCR

Leaf samples were collected on the last day of the cold treatment from the same leaf used for phenotypic measurements. We selected two groups of five accessions that were among the highest and lowest for *E* in the cold treatment, to test expression of three candidate genes (*Sb08g007310*, *Sb08g007340*, and *Sb08g016070*). Each group harbored a different allele of the significant SNP located close to or within the selected genes.

RNA extraction was performed using an RNeasy Mini Kit (QIAGEN, Valencia, CA, USA) and the RNA product was treated with DNase I to eliminate genomic DNA. RNA quality and quantity was tested with a NanoDrop spectrophotometer (NanoDrop Technologies, Wilmington, DE, USA). Reverse transcription–PCR (RT–PCR) was performed using SuperScript^®^ III reverse transcriptase for cDNA synthesis and the PCR product was run on a 1% agarose gel using SYBR Safe dye.

## Results

### Analysis of phenotypes

**Fig. 1.**
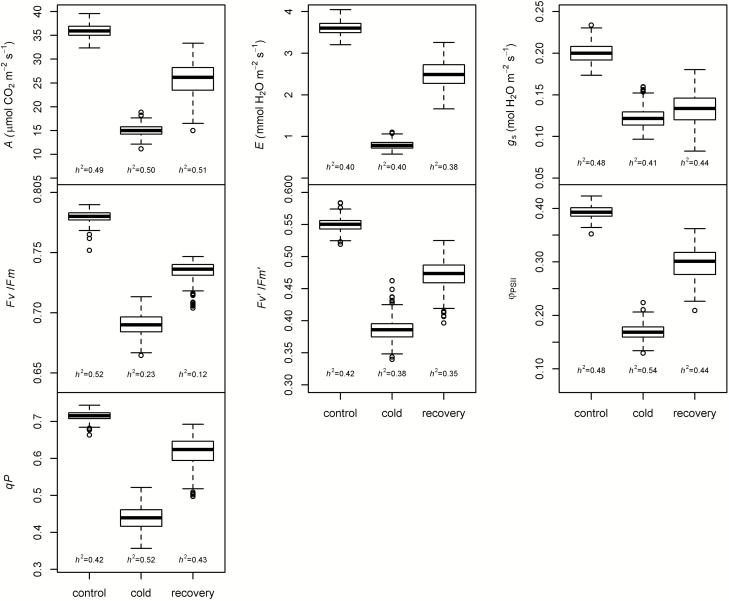
Box plots of gas exchange and chlorophyll fluorescence traits based on BLUPs for control, cold, and recovery periods. *h*^2^, heritability; *A*, photosynthesis; *E*, transpiration; *g*_*s*_, stomatal conductance; *F*_v_/*F*_m_, maximum quantum yield of PSII; Φ_PSII_, effective quantum yield of PSII; *F*_v_'/*F*_m_', efficiency of energy captured by open PSII reaction centers; and *qP*; photochemical quenching or fraction of PSII reaction centers that are open.

Significant variation was observed for all gas exchange and chlorophyll fluorescence variables (Fig. 1), but cold and recovery treatments generated larger ranges of variation than the control. Gas exchange variables decreased from control to cold periods more markedly (40–78%) than fluorescence variables (11–56%). Additionally, Φ_PSII_ and *qP* were the most affected fluorescence traits during the cold period (Supplementary Figs S3, S4). There were several significant correlations between variables in individual periods and when evaluated over treatments (Supplementary Tables S1–S6). *g*_s_ and *E* were correlated with almost all variables, which highlights the importance of stomatal effects on the physiological traits under investigation. *E*, *g*_*s*_, Φ_PSII_, and *qP* had the highest positive correlations with *A* (*P*<0.001).

As expected based on the general correlation trend, changes in *A* throughout temperature treatments were accompanied by changes in other gas exchange and fluorescence variables. While *A* and *g*_s_ decreased in cold and recovery treatments, reductions in *A* were proportionally larger in cold days at any *g*_s_ value (Supplementary Fig. S5).

The statistical analysis demonstrated that adequate variation for an association study exists in this panel since genotype (accession) was the only significant random effect for all traits (*P*<0.05), with variance estimates, in general, larger than set and replication nested in set (i.e. growth chamber effect; Supplementary Table S7). Among the fixed effects, day was significant for all variables (*P*<0.05) while machine was not significant for *E* recovery, *F*_v_'/*F*_m_' cold, *F*_v_'/*F*_m_' recovery, and Φ_PSII_ recovery. Heritability values were intermediate for most variables, ranging from 0.31 to 0.54, with low values observed only for *F*_v_/*F*_m_ cold and recovery (*h*^*2*^=0.23 and 0.12, respectively) (Fig. 1).

### Genome-wide association results

**Fig. 2.**
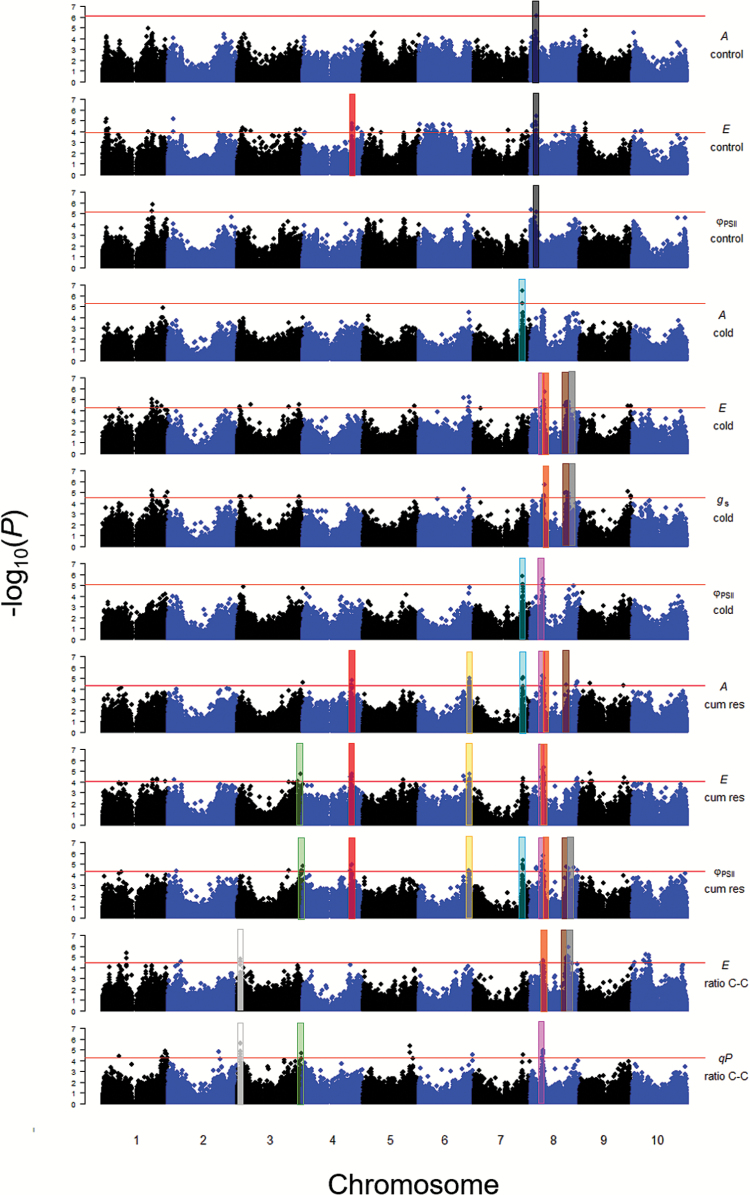
Most relevant genome-wide association results for photosynthesis and chlorophyll fluorescence for further validation studies. The horizontal red line indicates the significance threshold based on the false discovery rate. Each single nucleotide polymorphism is represented by a dot. Shaded colored areas highlight the most interesting genomic regions consistently identified associated with both gas exchange and chlorophyll fluorescence traits. Each color represents a unique region defined by LD levels and described in Table 2. Manhattan plots for *g*_s_ ratio control–cold, *g*_s_ cumulative response, *qP* cumulative response, and Φ_PSII_ ratio control–cold were not included in the figure for simplicity. *A*, photosynthesis (μmol CO_2_ m^–2^ s^–1^); *E*, transpiration rate (mmol H_2_O m^–2^ s^–1^); *g*_s_, stomatal conductance (mol H_2_O m^–2^ s^–1^); Φ_PSII_, effective quantum yield of PSII; *qP*, photochemical quenching or fraction of PSII reaction centers that are open; Cum res, cumulative response; ratio C–C, ratio control–cold.

Significant marker–trait associations were detected for seven traits throughout three individual temperature periods and for their derived variables cumulative response, ratio control–cold, and ratio cold–recovery (Table 1; Fig. 2; Supplementary Table S8; Supplementary Figs S6–S12). The number of significant markers varied between traits, from a minimum of one SNP for each of two variable–treatment combinations (*A* control and *qP* recovery), to many markers in all chromosomes for one parameter (*E* control). The percentage of the phenotypic variance explained by each marker (*R*^2^) ranged from 5.3% to 14%, with the highest *R*^2^ observed for marker *S6_47621281* in *F*_v_*/F*_m_ during the recovery treatment. No single SNP was coincidently associated with a trait in multiple temperature treatments, but marker *Sb03g004670* was significant in both control and cold for two highly correlated traits (*E* and *g*_s_). Additionally, in several cases, the same marker–trait association was detected for both an individual period and when treatments were integrated in a derived variable (Supplementary Table S8). For example, *S7_55202510* explained variation in *A* cold and cumulative response.

**Table 1.** Summary of significant SNPs associated with photosynthesis and chlorophyll fluorescence traits in three temperature periods

Trait	FDR threshold	Corresponding *P*-value	Chromosome	*R* ^2^ range	No. of significant SNP representative regions^*a*^
*A* control	0.095	7.93 × 10^–7^	8	(0.104–0.104)	1
*E* control	0.162	1.15 × 10^–4^	1–10	(0.053–0.115)	40
*g*_s_ control	0.096	3.31 × 10^–6^	1	(0.08–0.089)	2
*F*_v_'*/F*_m_' control	0.109	7.30 × 10^–5^	1, 2, 4, 5, 6, 8, 10	(0.056–0.108)	16
Φ_PSII_ control	0.155	6.87 × 10^–6^	1, 8	(0.082–0.099)	3
*qP* control	0.190	4.56 × 10^–6^	4	(0.081–0.081)	1
*A* cold	0.190	4.68 × 10^–6^	7	(0.076–0.092)	1
*E* cold	0.161	5.58 × 10^–5^	1, 3, 5, 6, 8, 9	(0.057–0.085)	19
*g*_s_ cold	0.132	3.07 × 10^–5^	1, 3, 6, 8, 9	(0.061–0.095)	17
Φ_PSII_ cold	0.159	8.04 × 10^–6^	7, 8	(0.072–0.083)	2
*qP* cold	0.192	3.10 × 10^–5^	2, 3, 4, 9	(0.06–0.1)	6
*A* recovery	0.138	2.40 × 10^–6^	4, 8	(0.099–0.105)	2
*F*_v_/*F*_m_ recovery	0.092	2.25 × 10^–6^	2, 4, 6	(0.088–0.14)	3
*qP* recovery	0.199	7.49 × 10^–5^	3	(0.116–0.116)	1
*A* cumulative response	0.176	4.79 × 10^–5^	3, 4, 6, 7, 8, 9	(0.058–0.1)	13
*E* cumulative response	0.176	9.09 × 10^–5^	1–9	(0.053–0.091)	19
*g*_s_ cumulative response	0.144	4.18 × 10^–5^	1, 3, 4, 5, 6, 7, 8, 9	(0.06–0.092)	18
Φ_PSII_ cumulative response	0.152	4.62 × 10^–5^	2, 3, 4, 6, 7, 8	(0.059–0.092)	14
*qP* cumulative response	0.078	7.08 × 10^–7^	1–10	(0.056–0.103)	21
*E* ratio control–cold	0.118	3.66 × 10^–5^	1, 2, 3, 8, 10	(0.06–0.104)	9
*g*_s_ ratio control–cold	0.113	4.04 × 10^–5^	1, 3, 4, 8, 9, 10	(0.061–0.129)	17
Φ_PSII_ ratio control–cold	0.189	1.95 × 10^–5^	1, 3, 6, 7, 8	(0.068–0.093)	7
Φ_PSII_ ratio cold–recovery	0.073	1.11 × 10^–6^	1	(0.086–0.09)	2
*qP* ratio control–cold	0.186	5.25 × 10^–5^	1, 2, 3, 5, 6, 7, 8	(0.06–0.099)	12

*A*, photosynthesis (μmol CO_2_ m^–2^ s^–1^); *E*, transpiration (mmol H_2_O m^–2^ s^–1^); *g*_*s*_, stomatal conductance (mol H_2_O m^–2^ s^–1^); *F*_v_/*F*_m_, maximum quantum yield of PSII; Φ_PSII_, effective quantum yield of PSII; *F*_v_'/*F*_m_', efficiency of energy captured by open PSII reaction centers; *qP*, photochemical quenching or fraction of PSII reaction centers that are open.

^*a*^ SNPs in physical proximity and in LD were considered a single region.

There were no significant marker–trait associations for the specific set of SNPs within or close (±5 kb) to the 67 *a priori* candidate genes (Supplementary Table S11; Supplementary Figs S6–S12), though in some cases the *q* values were close to the significance threshold.

In general, results for genome-wide markers could be summarized as those significant polymorphisms that were within genes with predicted functions related to photosynthesis or stress response and those SNPs physically close to candidate genes with a maximum distance of ~55 kb (Supplementary Tables S9, S10). Gene annotations of the former group include transcription factors, ATPase transporters, and genes related to carbon metabolism, while the second group include genes associated with photosynthetic processes, photosystem composition, and hormonal groups that determine plant responses to environmental stimulus. In both cases, these genes are suggested as good candidates for further validation experiments.

Considering the large number of significant regions identified, we proposed a strategy to prioritize these discoveries considering that those markers/chromosomal segments simultaneously associated with gas exchange and chlorophyll fluorescence traits represent the most promising and robust results for future validation. Using this prioritization criterion, we identified 10 regions on chromosomes 3, 4, 6, 7, and 8 that contained several SNPs in LD, and explained variation in one or more gas exchange traits and chlorophyll fluorescence parameters (Fig. 2; Table 2; Supplementary Figs S6–S12; Supplementary Table S9).

Three candidate genes (*Sb08g007310*, *Sb08g007340*, and *Sb08g016070*) were selected from these consistently significant regions, for further investigation based on the proximity or co-localization with significant SNPs, and on homology with photosynthesis and stress response genes. Expression of these three genes was tested in two groups of five accessions each, with the corresponding contrasting phenotypes, namely high and low *E* in the cold treatment (Supplementary Fig. S13). While there was no detectable expression of *Sb08g007340* and *Sb08g016070*, we found evidence of lower expression levels of *Sb08g007310*, a member of the glutathione *S*-transferase (GST) family, on leaves of accessions with low *E* in cold stress (Supplementary Fig. S13).

**Table 2.** Significant chromosomal regions delimited by multiple SNPs in linkage disequilibrium. Some SNPs in these regions were consistently controlling variation for multiple traits.

Chromosome	Physical position (start–end)^*a*^	Distance (kb)	Traits	No. of markers
3	3730025–3828318	98	*E* ratio control-cold, Φ_PSII_ ratio control–cold, *qP* ratio control–cold	10
3	72086161–72511657	425	*E* cum res, *g*_s_ cum res, Φ_PSII_ cum res, *qP* cum res, *qP* ratio control–cold	4
4	54825903–54871067	45	*A* cum res, *E* control, *E* cum res, *g*_s_ cum res, Φ_PSII_ cum res	6
6	57015199–57159773	145	*A* cum res, *E* cum res, Φ_PSII_ cum res	10
7	55198507–55202510	4	*A* cold, *A* cum res, Φ_PSII_ cold, Φ_PSII_ cum res, *qP* cum res	3
8	5297131–6112866	816	*A* control, *E* control, Φ_PSII_ control	3
8	12680072–14235598	1556	*A* cum res, *E* cold, *E* cum res, *g*_s_ cum res, Φ_PSII_ cold, Φ_PSII_ cum res, *qP* cum res, *qP* ratio control–cold, *g*_*s*_ ratio control–cold, *E* ratio control–cold	21
8	14499100–15326960	828	*A* cum res, *E* cold, *E* cum res, *g*_s_ cold, *g*_s_ cum res, Φ_PSII_ cum res	7
8	39996123–41851065	1855	*A* cum res, *E* cold, *g*_s_ cold, Φ_PSII_ cum res, *qP* cum res, *g*_s_ ratio control– cold, *E* ratio control–cold	6
8	42372182–42774340	402	*E* cold, *g*_s_ cold, Φ_PSII_ cum res, *g*_s_ ratio control–cold, *E* ratio control–cold	6

Cum res, cumulative response.

^*a*^ Physical position based on sorghum genome v1.4.

## Discussion

### Effect of control temperature on gas exchange and chlorophyll fluorescence traits

Gas exchange and chlorophyll fluorescence parameters presented a wide range of variation, suggesting that selection and genetic improvement of these traits are possible (Fracheboud *et al.*, 1999; Kościelniak *et al.*, 2005; Flood *et al.*, 2011). The range of variation in *A* values was similar to those previously reported in sorghum under optimal temperature conditions in the greenhouse (Peng and Krieg, 1992; Balota *et al.*, 2008; Xin *et al.*, 2009; Salas Fernandez *et al.*, 2015) and lower than maximum values in the field (Kidambi *et al.*, 1990; Peng and Krieg, 1992; Henderson *et al.*, 1998; Balota *et al.*, 2008). A comparative analysis of the photosynthetic rate of several accessions in common with Balota *et al.* (2008) and Salas Fernandez *et al.* (2015) demonstrated a consistent ranking of materials—Tx430 (PI 655996), SC265 (PI 533766), and SC56 (PI 533910) ranked among the highest in *A*, while El Mota (PI 656035) and Kuyuma (PI 656044) presented the lowest *A* values.

*F*_v_ */F*_m_ represents the maximum quantum yield of PSII (Maxwell and Johnson, 2000), and the observed values under control temperature agree with those reported in C_4_ species (Björkman and Demmig, 1987; Fracheboud *et al.*, 1999; Friesen *et al.*, 2014) including sorghum (Cousins *et al.*, 2002).

Characterizing the photosynthetic performance of a large number of accessions is a challenging task, given the multiple factors that can affect data accuracy and precision. For instance, the photosynthetic rate of a genotype can be affected by the age of the leaf, changes throughout the day, and plant to plant variation. The intermediate *h*^2^ observed in our study for most variables, similar to those reported for other species (Hervé *et al.*, 2001; Fracheboud *et al.*, 2004; Gu *et al.*, 2012*b*), are evidence of these non-genetic effects and the physiological and genetic complexity of these traits. Therefore, it is not surprising that direct breeding efforts to obtain higher photosynthetic performance have been elusive in the past (Crosbie and Pearce, 1982; Flood *et al.*, 2011).

### Effect of cold and recovery treatments on gas exchange and chlorophyll fluorescence traits

Cold and the subsequent recovery treatments significantly reduced all traits, in agreement with previous reports for C_4_ species (Fracheboud *et al.*, 1999; Kościelniak *et al.*, 2005; Fiedler *et al.*, 2012; Bekele *et al.*, 2014). In our study, plants were grown under optimal conditions and subsequently exposed to low temperature, which resembles a cold event during spring. This contrasts with other reports in maize (Kościelniak *et al.*, 2005) and sorghum (Bekele *et al.*, 2014; Fiedler *et al.*, 2014, 2016), in which plants were grown under suboptimal temperatures throughout the experiment. The physiological mechanisms explaining contrasting genotypes are different in each case, since in our study both direct cold responses and cold acclimation/adaptation probably played an important role in determining the genotype-specific performance.

The decrease in *A* was accompanied by lower *g*_s_ under cold and recovery treatments, but at any given *g*_s_ value, *A* was lower in the cold than in the recovery period (Supplementary Fig. S5), confirming that, as reported in maize (Nie *et al.*, 1992; Fracheboud *et al.*, 2002), stomata were not the only factor limiting *A* at low temperature.

The lower Φ_PSII_ under cold stress indicates a reduction in the rate of linear electron transport, a phenomenon also reported in maize (Fracheboud *et al.*, 1999; Kościelniak *et al.*, 2005) and miscanthus (Friesen *et al.*, 2014). The observed simultaneous decrease in *F*_v_'/*F*_m_' and *qP* suggests some level of stress-induced saturation and photodamage. Fracheboud *et al.* (1999) reported similar results in maize grown under optimal conditions and exposed to cold stress. However, when constantly grown under suboptimal conditions, lower Φ_PSII_ values were associated mostly with decreases in *F*_v_'/*F*_m_' (Fracheboud *et al.*, 1999; Kościelniak *et al.*, 2005).

The reductions in *F*_v_'/*F*_m_' in the cold and recovery treatment are evidence of an increase in non-photochemical quenching. Under cold stress, plants experience ∆pH-dependent conformational changes in the PSII antenna associated with PsbS protein and the xanthophyll cycle, which increases the energy dissipation capacity as heat, protecting PSII but reducing the utilization of light energy in photochemical processes (Adams *et al.*, 2006; Ruban *et al.*, 2012). Additionally, the lower *F*_v_*/F*_m_ observed during cold and recovery days can be related to a degree of photodamage in the plants (Maxwell and Johnson, 2000), and the retention of xanthophyll cycle carotenoids as zeaxanthin and antheraxanthin (Adams *et al.*, 2006).

### Genomic regions associated with gas exchange and chlorophyll fluorescence traits

A set of 10 regions was consistently associated with both gas exchange and chlorophyll fluorescence parameters and, considering the methodological differences between these two groups of traits, these markers on chromosomes 3, 4, 6, 7, and 8 (highlighted in Fig. 2; Table 2) represent the most promising discoveries for future validation studies. Candidate genes localized within 55 kb of the significant polymorphisms (Supplementary Table S9) have predicted functions related to photosynthesis, carbon, and nitrogen metabolism, transcription factors, protein kinases, transporters, and several unknown proteins.

An additional 133 markers/regions significantly associated with the traits of interest represent unique discoveries for a single variable or for multiple but derived traits (e.g. cold and cumulative response). These polymorphisms were physically close to a set of 99 candidate genes with diverse functions such as carotenoids, phytohormones, and components of PSI and PSII, among others (Supplementary Table S10). We present herein the most important SNPs/genomic regions and promising candidate genes.

#### For control temperature

We identified 60 SNPs/genomic regions associated with one or more traits in the control treatment, and one of these regions included SNPs for both gas exchange and chlorophyll fluorescence traits, namely *A* control, *E* control, and Φ_PSII_ control (Table 2; Fig. 2; Supplementary Table S9).

Considering the multiple processes involved in photosynthesis, we hypothesized that natural photosynthetic variation in sorghum is controlled by genes related to stomatal limitations, Calvin cycle, and the light reactions. Our association results and their corresponding candidate genes support this hypothesis. For example, the observed high correlations between *A*, *E*, and *g*_s_ imply that the regulation of stomatal aperture affected *A* in the control period and is supported by the seven markers within the *Abscisic aldehyde oxidase* gene (*AAO*, *Sb01g005650*) that were associated with *E* control. Given the role of ABA in the regulation of stomatal aperture in response to environmental factors (Zhang and Davies, 1990; Trejo *et al.*, 1995), the observed phenotypic variation in *E,* and thus *A*, could be related to differences in abscisic acid (ABA) content in leaves (Conti *et al.*, 1994).

The genotypic differences in Φ_PSII_ are explained by variations in energy capture (*F*_v_'/*F*_m_') and utilization (*qP*). Interestingly, the low correlation between the latter variables (Supplementary Table S1) suggests that different mechanisms control these two processes. The efficiency of the light reactions could be affected by the carotenoid content in leaves, given their role in energy dissipation through the xanthophyll cycle, as pigments of the photosystem complexes and antioxidants (Müller *et al.*, 2001). Supporting this hypothesis, three candidate genes related to carotenoid biosynthesis were discovered close to significant markers: *Phytochrome interacting factor 4* (*PIF4*, *Sb08g019780* and *Sb08g021000*) in *F*_v_'/*F*_m_', and *1-deoxy*-*d**-xylulose 5-phosphate reductoisomerase* (*DXR*, *Sb03g008650*) in *E*. Two of them (*Sb08g019780* and *Sb03g008650*) were *a priori* candidates, but markers within or close (±5 kb) to them were non-significant (as described above). However, these gene-specific markers were located on introns, downstream, or represented synonymous polymorphisms, and,thus, considering that distant significant markers (in this case within 30.8 kb) could be in LD with a causal polymorphism, we still consider these genes important for further investigation.

Differences in carbon fixation reactions could also be the underlying mechanism causing natural variation in photosynthesis. The two thioredoxin genes, *Sb03g004670* and *Sb06g029490*, close to significant markers in *E* and *F*_v_'/*F*_m_', support this hypothesis since they are related to light-dependent activation of chloroplastic enzymes in the Calvin cycle (Okegawa and Motohashi, 2015).

Finally, genes of potential interest for further studies include several peroxidases, one ATP synthase (*Sb01g042930*), and a component of LHCI (*Sb02g037410*) (Supplementary Tables S9, S10).

#### For cold and recovery

A total of 37 SNPs/genomic regions were associated with one or more traits in the cold and recovery treatments, including five on chromosomes 7 and 8 that were significant for both gas exchange and chlorophyll fluorescence traits (Fig. 2; Table 2).

Plants exposed to cold respond with a cascade of changes in gene expression regulated by transcription factors designated CBF, inducer of C-repeat binding factor (Chinnusamy *et al.*, 2007). Therefore, we hypothesize that transcriptional changes leading to cold acclimation are the main cause of differential photosynthetic responses under cold. Some evidence supporting this idea includes the *APETALA2* transcription factor gene (*Sb09g024400*) identified close to a marker significant for *E* and *g*_s_. Members of this gene family are involved in the acclimation process and were differentially expressed in sorghum in response to cold (Chopra *et al.*, 2015).

Multiple genes involved in oxidative stress, including peroxidases, *Vitamin C deficient 2* (*VTC2*, *Sb03g042900*), and heat shock proteins that act as protein chaperons in response to stress (Feder and Hofmann, 1999), were predicted close to associated markers. This evidence suggests that stress response genes are important for the sorghum photosynthetic response to cold. Most importantly, two *GST* genes (*Sb08g007310* and *Sb08g007340*) were close to markers in one selected genomic region (Table 2). *GST* genes encode a family of enzymes involved in herbicide detoxification, secondary metabolism, and as peroxidases under cold conditions (Roxas *et al.*, 1997). Considering the increased transcription of this gene family in response to stress (Glombitza *et al.*, 2004; Chopra *et al.*, 2015) and the differential expression of *GST* paralogs in sorghum accessions under cold (Chopra *et al.*, 2015), the transcription of these two *GST* genes was investigated herein. This preliminary study confirmed the differential expression of *Sb08g007310* in 10 lines with extreme levels of *E* under cold stress (Supplementary Fig. S13). Additionally, we identified multiple *Cytochrome P450* genes (*CYP*), members of the monooxygenase superfamily that catalyze oxidative reactions (Isin and Guengerich, 2007) and are involved in the biosynthesis of phytohormones and secondary metabolites (Morant *et al.*, 2003). Differential expression of *CYP* genes has been reported in response to abiotic and biotic stress (Narusaka *et al.*, 2004; Gelli *et al.*, 2014), including cold in sorghum (Chopra *et al.*, 2015).

In miscanthus, a cold-tolerant C_4_ species, acclimation to low temperature conditions is mostly due to the stability of the *PPDK*-encoded enzyme (Wang *et al.*, 2008). We did not find evidence that genes related to the C_4_ cycle are associated with sorghum response to cold. However, *Phosphoenolpyruvate carboxylase kinase* (*Sb04g028135*) is localized close to a marker significant for *F*_v_/*F*_m_ recovery, and the encoded enzyme is known to regulate the phosphorylation of phosphoenolpyruvate carboxylase (Bakrim *et al.*, 1993) and the response of C_4_ species to cold (Matsuba *et al.*, 1997). Additionally, a significant marker for *g*_s_ in cold is close to the same thioredoxin gene found in the control period (*Sb03g004670*), suggesting that the regulation of Calvin cycle enzymes is important under both temperature conditions.

Three SNPs associated with *A* and Φ_PSII_ in cold are 1–5 kb from *Light-harvesting chlorophyll-protein complex I subunit A4* (*LHCA4*, *Sb07g021260*) (Supplementary Table S9), predicted to encode one of the four PSI polypeptides. Evidence suggests that LHCI can be affected by excessive light, particularly under cold stress (Terashima *et al.*, 1994; Scheller and Haldrup, 2005).

The reductions in *F*_v_'/*F*_m_' and *F*_v_*/F*_m_ during cold and recovery days can be explained by an increase in non-photochemical quenching and photodamage, respectively (Adams *et al.*, 2006; Ruban *et al.*, 2012). The discovery of a marker close to *Zeaxanthin epoxidase* (*ZEP*, *Sb06g018220*) in *F*_v_/*F*_m_ supports this idea. This gene is responsible for the epoxidation of zeaxanthin in the xanthophyll cycle, which is associated with the photosynthetic efficiency under cold and contrasting light conditions (Demmig-Adams and Adams, 1994; Fracheboud *et al.*, 2002).

#### For cumulative response and ratios

The cumulative response, ratio control–cold, and ratio cold–recovery variables facilitated the discovery of 82 significant SNPs/genomic regions. Markers consistently associated with both gas exchange and chlorophyll fluorescence traits were localized on chromosomes 3, 4, 6, 7, and 8 (Table 2). Some polymorphisms were discovered for both a derived variable and a temperature treatment, while several association were exclusively detected for these derived parameters (e.g. regions on chromosome 3 and 6; Table 2).

Cumulative response captures the overall performance and is affected by the multiple mechanisms triggered over the three temperature periods. Therefore, the 37 genomic regions uniquely identified in cumulative response traits are expected to control multiple processes. Candidate genes within these regions are related to response to stress (i.e. heat shock proteins, *APETALA2* transcription factors, and peroxidase), components of LHCI and LCHII, thioredoxin, transporters, and phytohormones (i.e. gibberellic acid and auxins) (Table 2; Supplementary Tables S9, S10).

Ratio variables compare the accessions using relative units, providing information about the sensitivity to cold and recovery periods. The most important genes close to the 17 genomic regions uniquely identified by ratio variables are related to response to stress (heat shock proteins, *CYP*), a component of LHCII, and phytohormones (Table 2; Supplementary Tables S9, S10).

### Comparison between our GWA results and previous studies

QTL studies for gas exchange and chlorophyll fluorescence under optimal and stress conditions have been reported in rice (*Oryza sativa*) (Gu *et al.*, 2012*b*), Arabidopsis (*Arabidopsis thaliana*) (Jung and Niyogi, 2009), maize (Fracheboud *et al.*, 2004; Yin *et al.*, 2015), and sunflower (*Helianthus annuus* L.) (Hervé *et al.*, 2001; Kiani *et al.*, 2008). Additionally, a few GWAS have been conducted under stress and non-stress conditions in maize (Strigens *et al.*, 2013), sorghum (Fiedler *et al.*, 2014, 2016), Arabidopsis (van Rooijen *et al.*, 2015), and soybean (*Glycine max*) (Hao *et al.*, 2012) for some of the physiological parameters investigated herein. In all cases, photosynthetic traits were associated with multiple genomic regions. In our study, the proportion of variation explained by each marker (5.3–14%) was within range of other GWAS results and, as expected, lower than QTL mapping findings (7–62%). Even though no markers were identified controlling a trait under both non-stress and stress conditions, several polymorphisms were associated with multiple parameters, an observation reported in other species (Fracheboud *et al.*, 2002; Kiani *et al.*, 2008; Yin *et al.*, 2015) that can be attributed to pleiotropy and the high correlations between variables.

In sorghum, several QTLs were reported for germination-related traits under cold stress (Yu and Tuinstra, 2001; Knoll *et al.*, 2008; Burow *et al.*, 2010; Bekele *et al.*, 2014), but the physiological mechanisms involved in those stress responses are probably different from those investigated in our study, considering the contrasting developmental stages and that, in our experiments, plants were grown under optimal temperatures before imposing the cold stress.

Two previous sorghum GWAS that evaluated chlorophyll fluorescence and growth traits discovered markers associated with *F*_v_'/*F*_m_' and Φ_PSII_ when plants were grown under suboptimal cold conditions (Fiedler *et al.*, 2014, 2016). In a comparative analysis, we identified a region on chromosome 4 that was 28–80 kb from a marker previously associated with *F*_v_'/*F*_m_' and Φ_PSII_ in response to cold (*sPb-3838*) (Fiedler *et al.*, 2014). We also discovered multiple SNPs close to markers previously reported by Fiedler *et al.* (2016): (i) *S6_50595426*, significant for *E* and *g*_s_ cumulative response is 15 kb from *UGSDI_27967* associated with *F*_v_'/*F*_m_'; (ii) *S8_54340453* that explained variation in Φ_PSII_ ratio control–cold, is 32 kb from *UGSS_05729*, also associated with Φ_PSII_; and (iii) *S8_48791253* and *S8_48794253,* significant for *qP* and Φ_PSII_ cumulative response, respectively, are 4 kb and 7 kb from *UGSS_05515* consistently significant for Φ_PSII_.

## Concluding remarks

This work aimed to characterize the natural genetic variation in photosynthetic rate and chlorophyll fluorescence in sorghum under non-stress, cold stress, and recovery conditions. The strength of our results resides in the large number of accessions and SNPs evaluated to investigate complex physiological traits, which resulted in a GWAS with good genome coverage and statistical power. This study provides new evidence to demonstrate that: (i) there is significant natural variation in the photosynthetic response of sorghum lines to cold stress and in their capacity to recover from that stress; (ii) sorghum lines also exhibit different carbon fixation capacities and chlorophyll fluorescence parameters under non-stress conditions; (iii) the genetic architecture of the differential photosynthetic response is complex for some traits (40 genomic regions for *E* under non-stress) and simple for others (one region for *qP* under control conditions); (iv) each individual marker/genomic region explained 5.3–14% of the phenotypic variance; (v) several candidate genes were identified co-localizing with significant markers or nearby; (vi) the predicted function of those genes suggests that they are related to carotenoids, phytohormones, components of PSI and PSII, antioxidants, and oxidative stress response; and (vii) the regions/markers on chromosome 3, 4, 6, 7, and 8, consistently identified for gas exchange and chlorophyll fluorescence traits, are the most important for further investigation. This new knowledge could be utilized after validation, to develop sorghum germplasm with superior carbon fixation capacity under cold conditions, which is necessary to increase biomass production and yield in temperate regions. Additionally, our understanding of the allelic variants associated with these physiological traits could help identify key processes and genes to manipulate by breeding or engineering approaches, to increase the photosynthetic capacity of other economically important crop species such as maize, rice and wheat.

## Supplementary data

Supplementary data are available at *JXB* online.

Fig. S1. Graphical representation of temperature treatments and days of phenotypic measurements.

Fig. S2. Graphical representation of the cumulative response calculation as the area under the curve.

Fig. S3. Photosynthesis (*A*) as a function of effective quantum yield of PSII (Φ_PSII_).

Fig. S4. Photosynthesis (*A*) as a function of photochemical quenching (*qP*).

Fig. S5. Photosynthesis (*A*) as a function of stomatal conductance (*g*_s_).

Figs S6–S12. GWAS results for photosynthesis and chlorophyll fluorescence traits.

Fig. S13. RT–PCR expression levels of the GST gene *Sb08g007310* using accessions with low and high *E* during the cold temperature treatment.

Tables S1–S6. Phenotypic correlations in control, cold, and recovery periods, and in cumulative response, ratio control–cold, and ratio cold–recovery.

Table S7. ANOVA of photosynthetic and chlorophyll fluorescence traits.

Table S8. Summary of GWAS results for photosynthesis and chlorophyll fluorescence traits in individual treatments, and the derived variables cumulative response, ratio control–cold, and ratio cold–recovery.

Table S9. Complete list of sorghum candidate genes close to (±55 kb) significant SNPs simultaneously associated with gas exchange and chlorophyll fluorescence traits.

Table S10. Candidate genes within 55 kb from significant SNPs for regions other than those described in Supplementary Table S9.

Table S11. *A priori* candidate genes and the corresponding SNPs within the gene or 5 kb upstream/downstream.

## Supplementary Material

supplementary_figures

supplementary_tables_S1_S7

supplementary_table_S10

supplementary_table_S11

supplementary_table_S8

supplementary_table_S9

## Abbreviations:

A: leaf carbon assimilation rate
CEF: cyclic electron flow around PSI
E: transpiration rate
*F*_v_/*F*_m_: maximum quantum yield of PSII
*F*_v_'/*F*_m_': efficiency of energy captured by open PSII reaction centers
GBS: genotyping-by-sequencing
*g*_s_: stomatal conductance
GWAS: genome-wide association study
LD: linkage disequilibrium
LHCI: light-harvesting complex I
LHCII: light-harvesting complex II
qP: photochemical quenching or fraction of PSII reaction centers that are open;
QTL: quantitative trait locus
SNP: single nucleotide polymorphism
Φ_PSII_: effective quantum yield of PSII.
