# Supplementary material for: Genetic architecture of photosynthesis in Sorghum bicolor under non-stress and cold stress conditions
Source: J Exp Bot. 2017 Aug 18;68(16):4545–57. doi: 10.1093/jxb/erx276 (PMC5853419; doi:10.1093/jxb/erx276)
Supplement: supplementary_figures [file erx276_suppl_supplementary_figures.pdf]

Genetic architecture of photosynthesis in *Sorghum bicolor* under non-stress and cold stress conditions

Diego Ortiz, Jieyun Hu, Maria G. Salas Fernandez

Supplementary figures

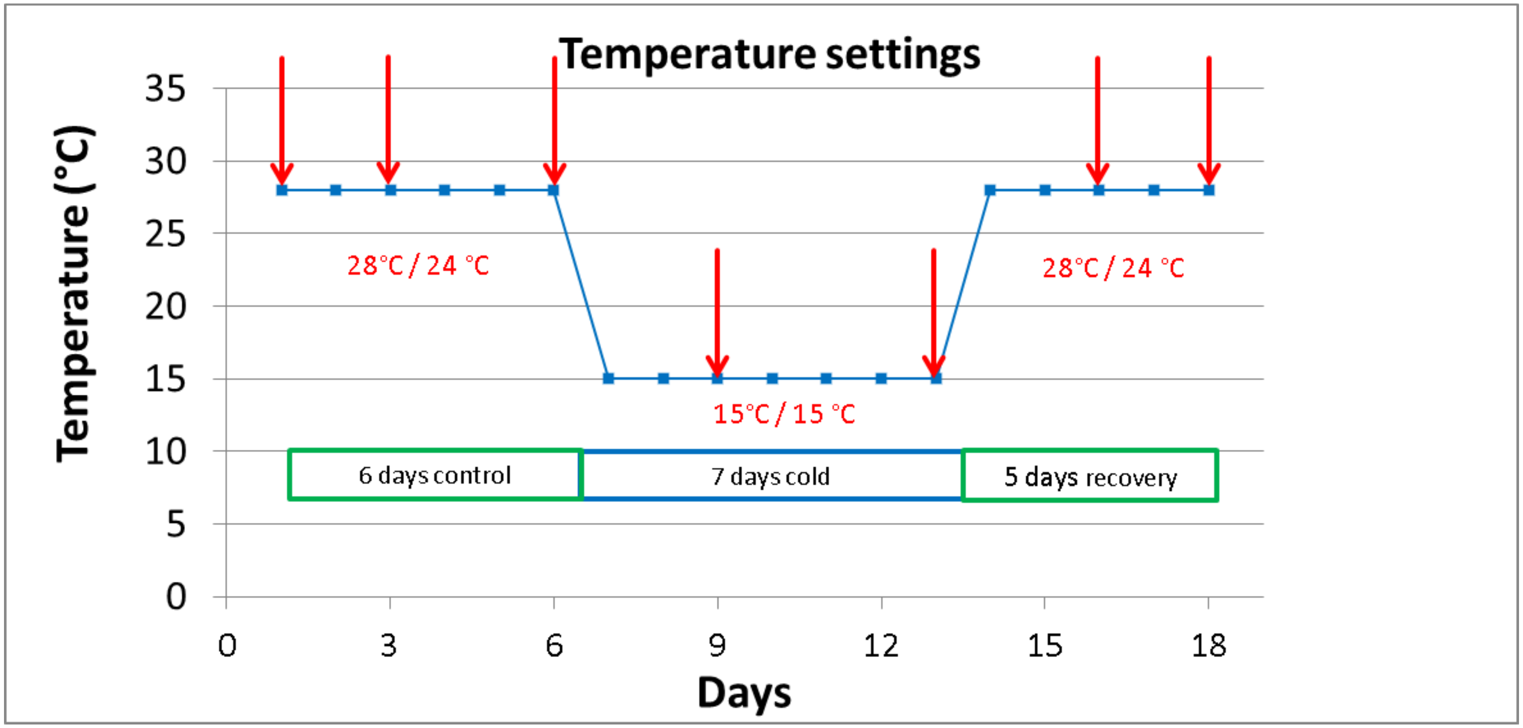

Supplementary Figure S1. Graphical representation of temperature treatments (control, cold and recovery periods). Day/Night temperatures are highlighted in red for each period. Arrows indicate the days of phenotypic measurements.

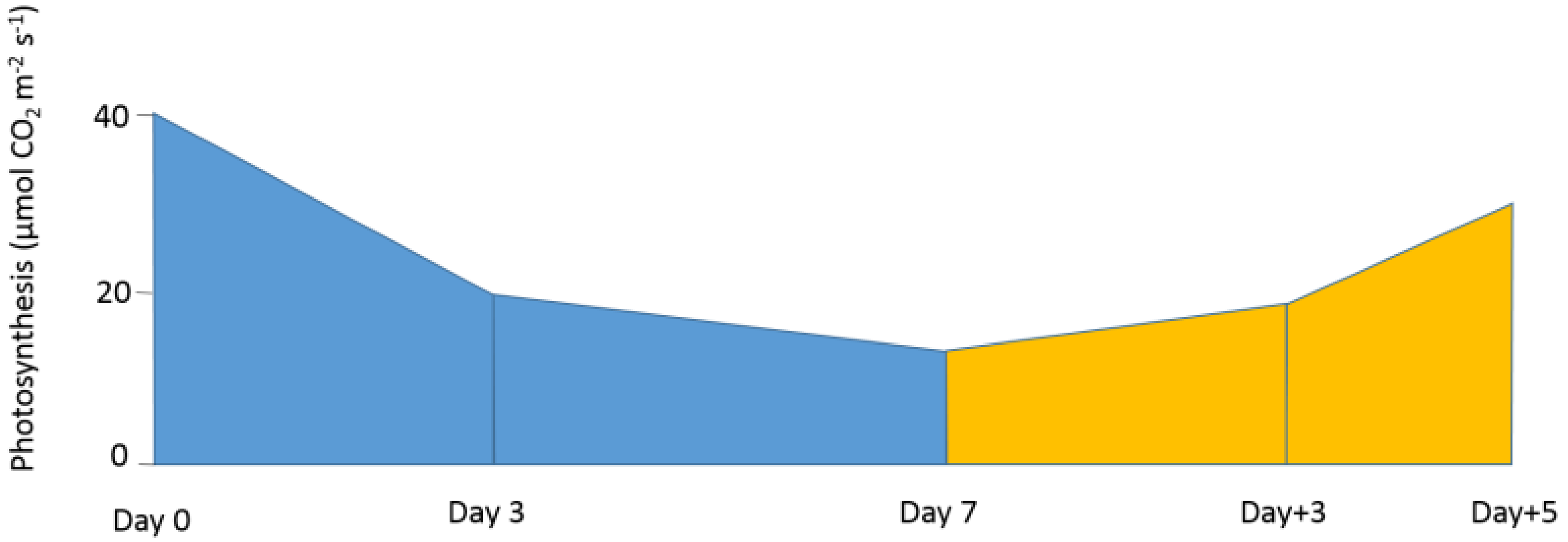

Supplementary Figure S2. Graphical representation for the calculation of cumulative response over the three temperature treatment periods. The same calculation was performed for all variables. Partial areas between days were calculated and summed up for estimating the total value. Day 0 represents the trait average value for the control period; days 3 and 7 (blue areas) contain values for the cold treatment and days +3 and +5 (orange areas) represent values in the recovery treatment.

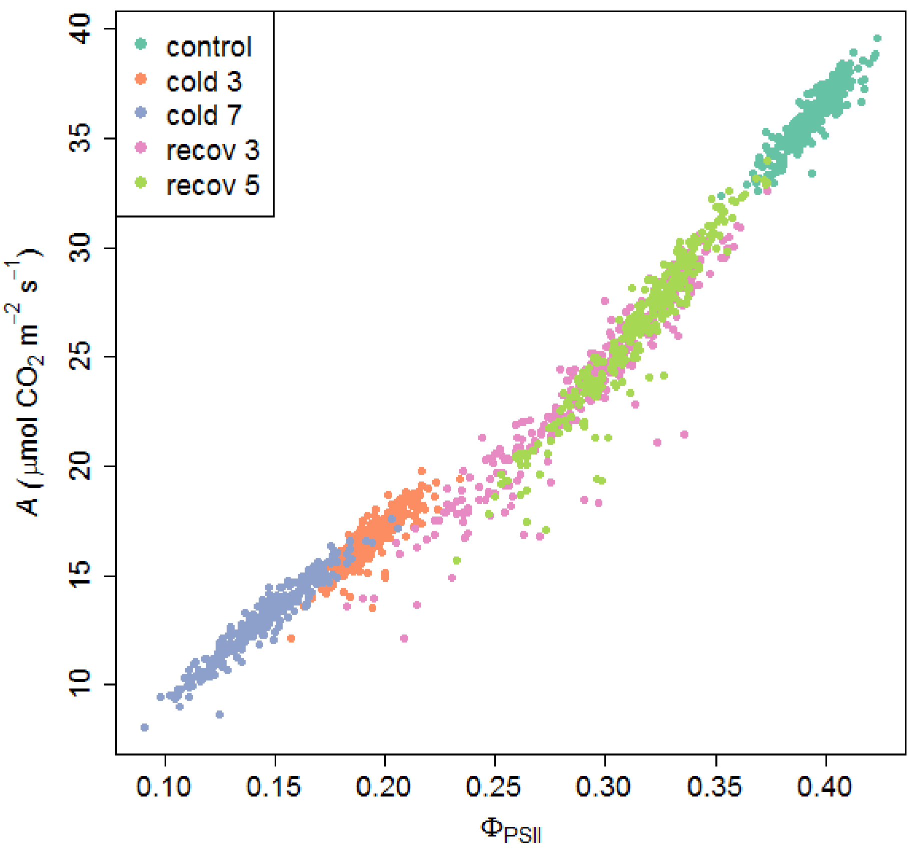

Supplementary Figure S3. Photosynthesis (*A*) as a function of effective quantum yield of PSII (Φ_{PSII}) in sorghum in control (28°C/24°C), cold (15°C/15°C), and recovery periods (28°C/24°C). All measurements were performed on fully expanded leaves from 30-day old plants grown in 6-L pots. Control = average of 3 days in the control treatment; cold 3 = day 3 of cold treatment; cold 7 = day 7 of cold treatment; recov 3 = day 3 of recovery period treatment; recov 5 = day 5 of recovery treatment.

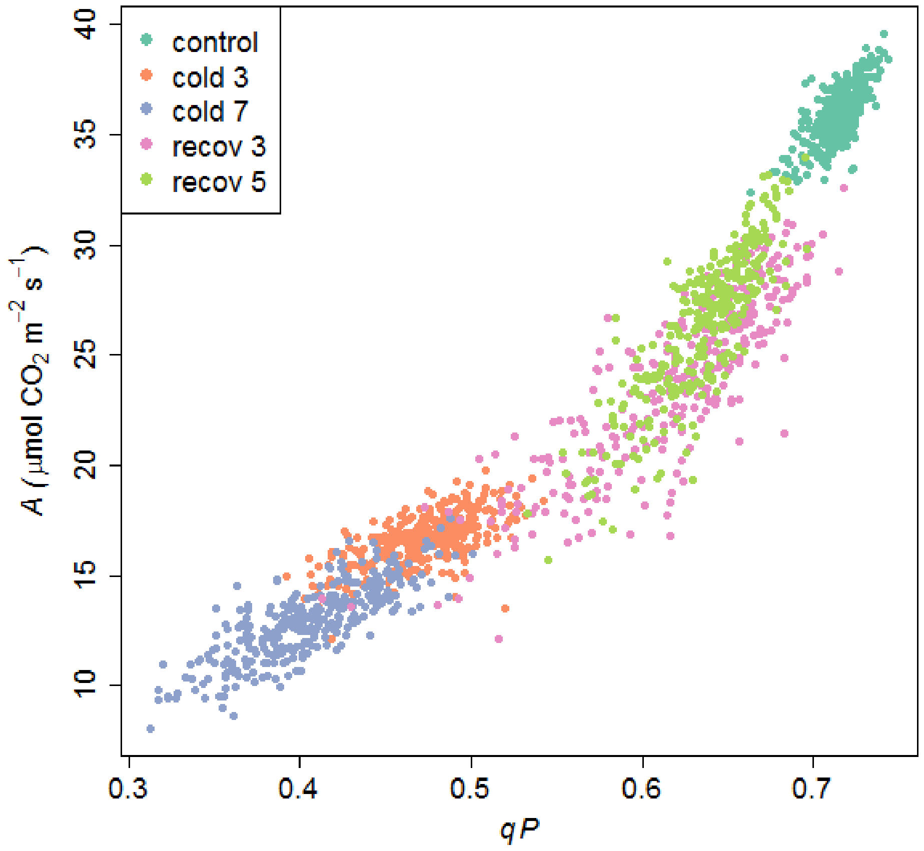

Supplementary Figure S4. Photosynthesis (*A*) as a function of photochemical quenching *(qP)* in sorghum in control (28°C/24°C), cold (15°C/15°C), and recovery periods (28°C/24°C). All measurements were performed on fully expanded leaves from 30-day old plants grown in 6-L pots. Control = average of 3 days in the control treatment; cold 3 = day 3 of cold treatment; cold 7 = day 7 of cold treatment; recov 3 = day 3 of recovery period treatment; recov 5 = day 5 of recovery treatment.

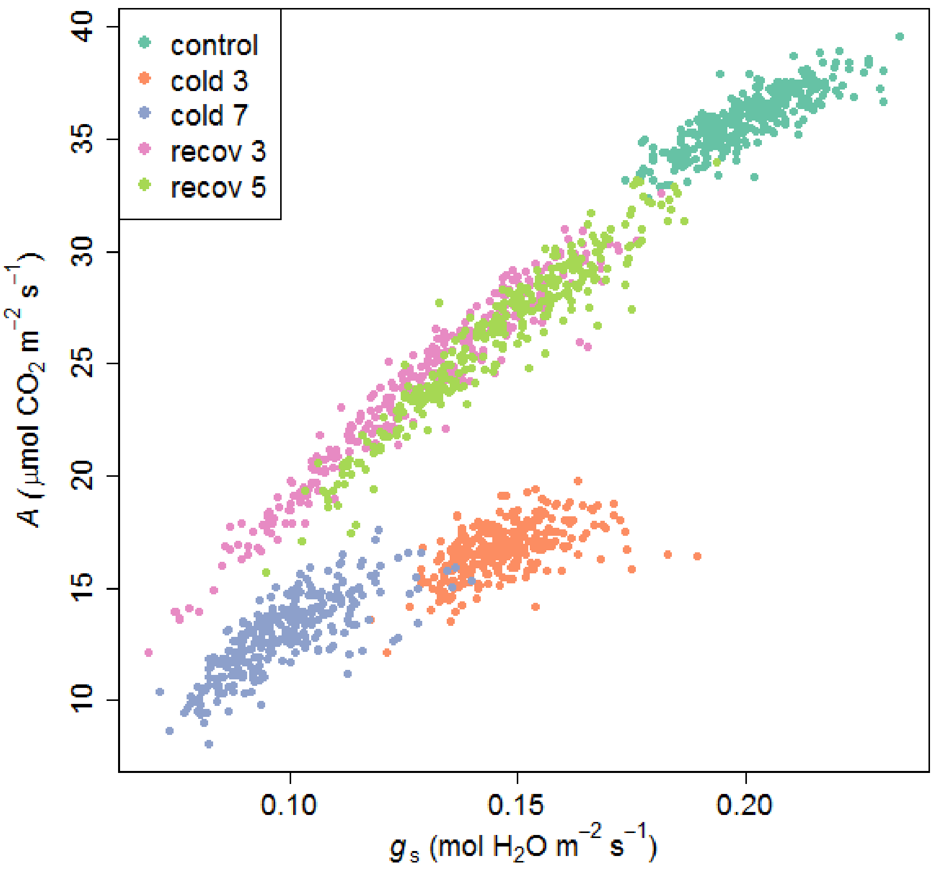

Supplementary Figure S5. Photosynthesis (*A*) as a function of stomatal conductance (g_s) in sorghum in control (28°C/24°C), cold (15°C/15°C), and recovery periods (28°C/24°C). All measurements were performed on fully expanded leaves from 30-day old plants grown in 6-L pots. Control = average of 3 days in the control treatment; cold 3 = day 3 of cold treatment; cold 7 = day 7 of cold treatment; recov 3 = day 3 of recovery period treatment; recov 5 = day 5 of recovery treatment.

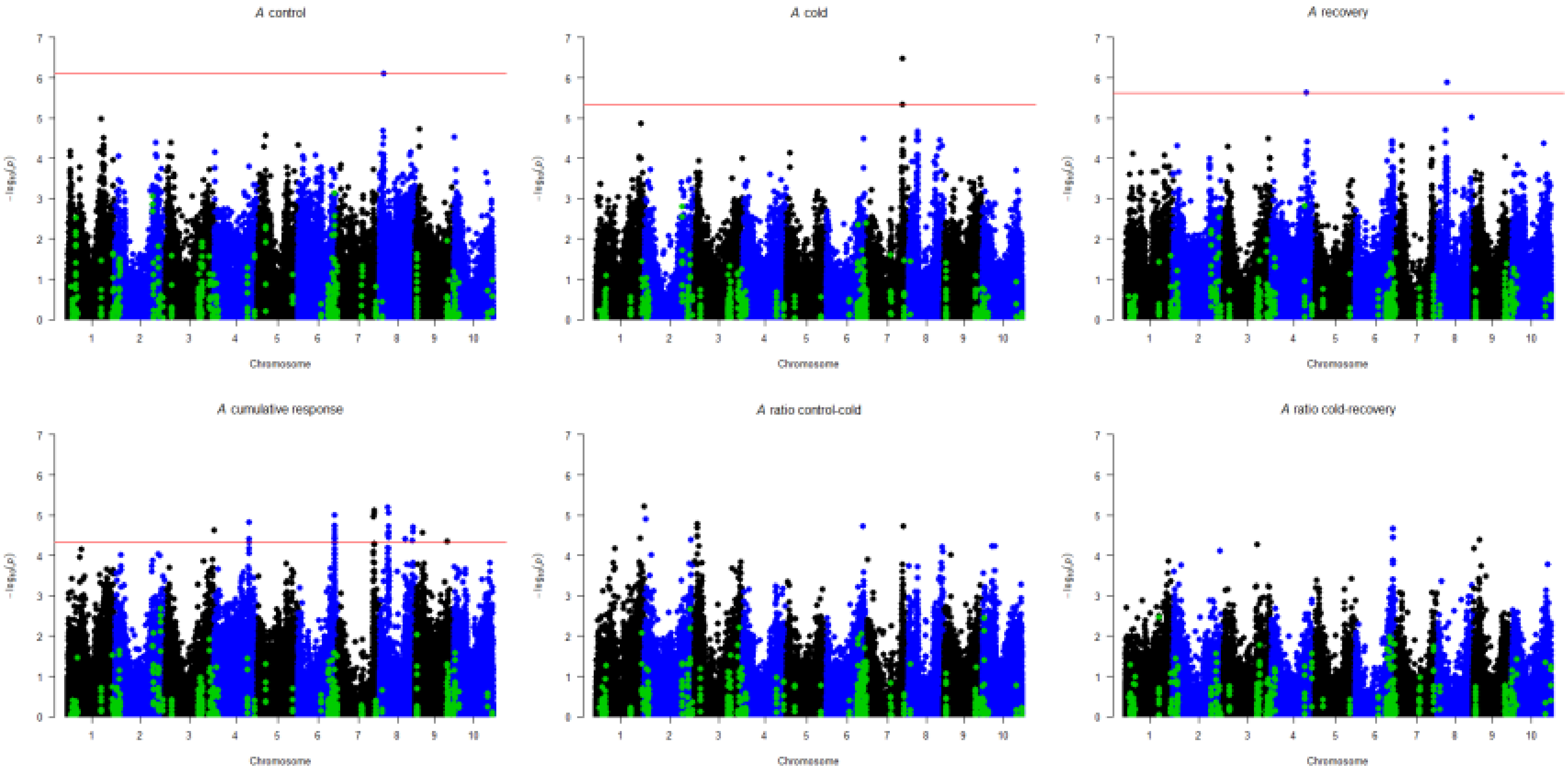

Supplementary Figure S6. Genome-wide association study results for *A* in three temperature treatments and in response area using 304 diverse sorghum accessions. Horizontal red line indicates significance threshold. Green dots indicate the physical position of *a priori* candidate genes. Each single-nucleotide polymorphism is represented by a dot, whose center indicates the exact physical position of the marker.

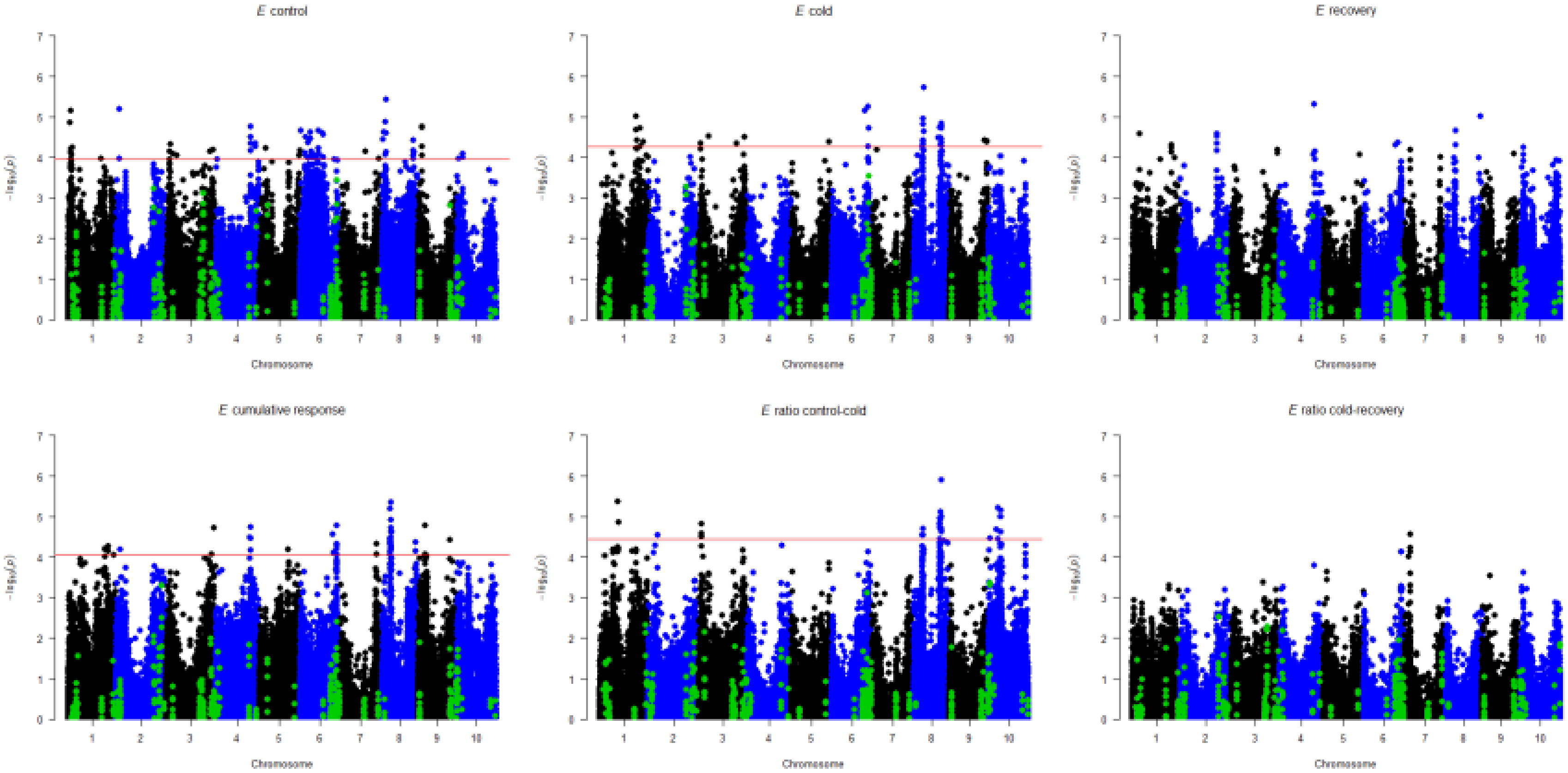

Supplementary Figure S7. Genome-wide association study results for *E* in three temperature treatments and in response area using 304 diverse sorghum accessions. Horizontal red line indicates significance threshold. Green dots indicate the physical position of *a priori* candidate genes. Each single-nucleotide polymorphism is represented by a dot, whose center indicates the exact physical position of the marker.

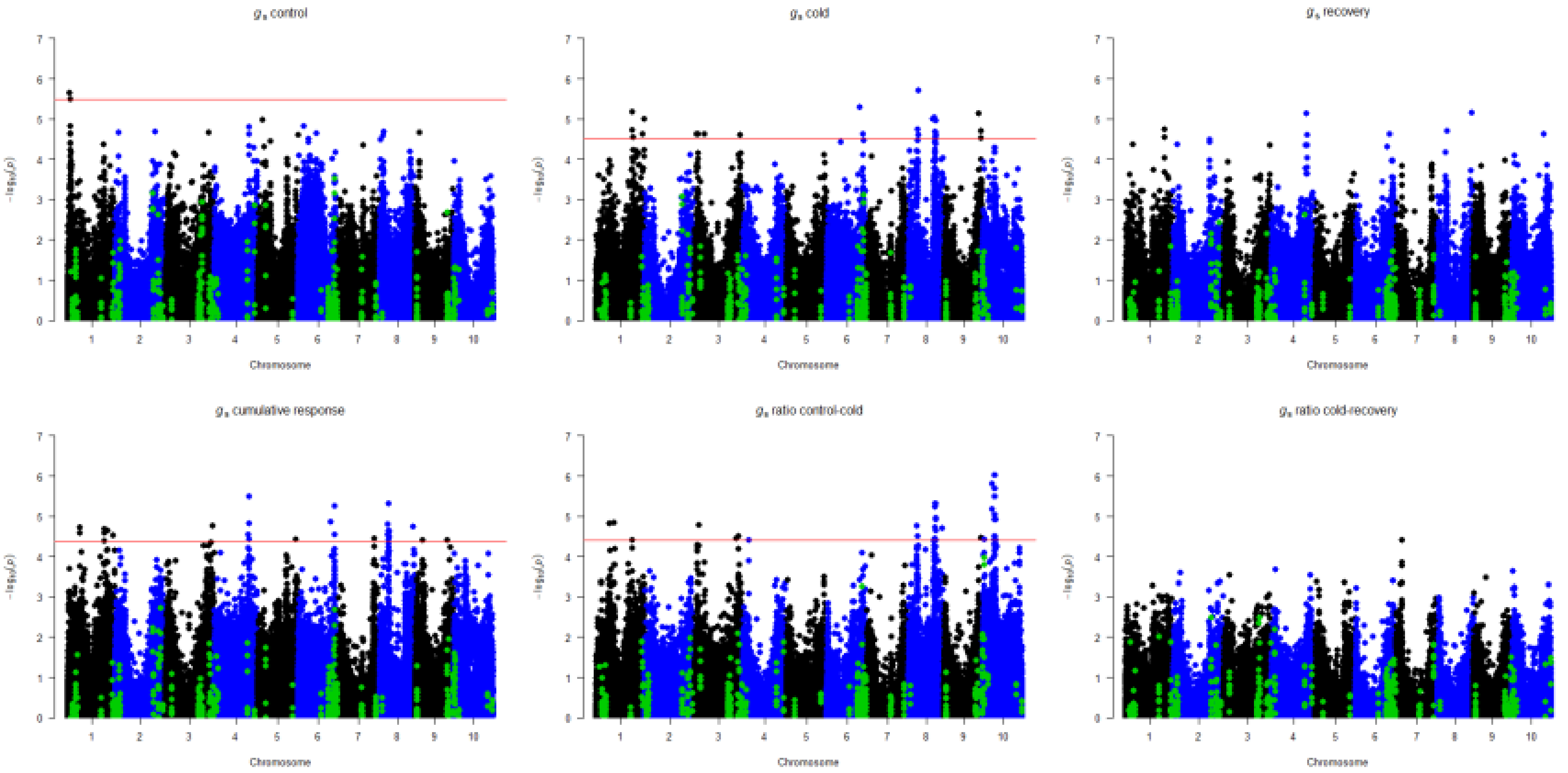

Supplementary Figure S8. Genome-wide association study results for g_s in three temperature treatments and in response area using 304 diverse sorghum accessions. Horizontal red line indicates significance threshold. Green dots indicate the physical position of *a priori* candidate genes. Each single-nucleotide polymorphism is represented by a dot, whose center indicates the exact physical position of the marker.

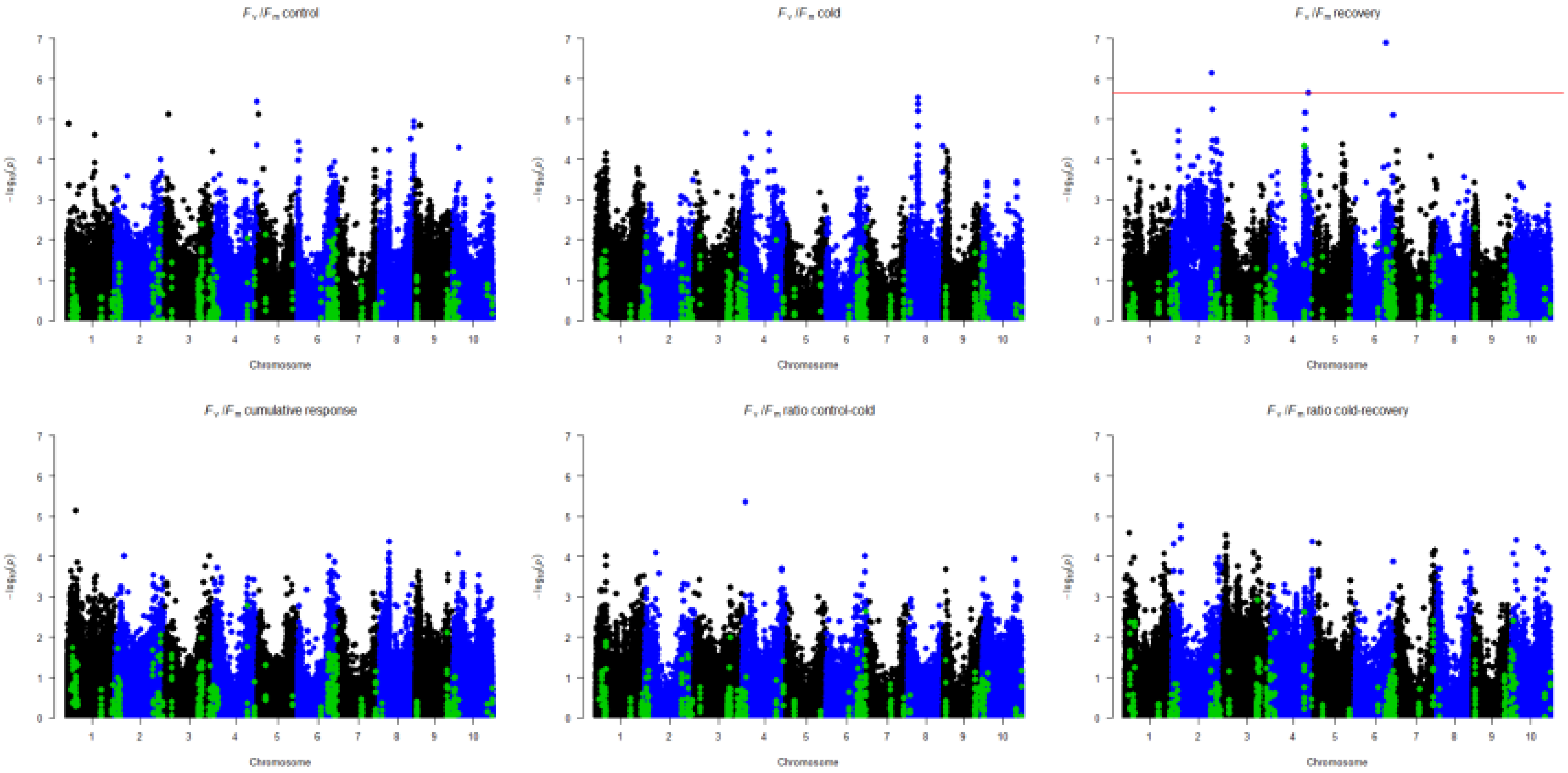

Supplementary Figure S9. Genome-wide association study results for F_v/F_m in three temperature treatments and in response area using 304 diverse sorghum accessions. Horizontal red line indicates significance threshold. Green dots indicate the physical position of *a priori* candidate genes. Each single-nucleotide polymorphism is represented by a dot, whose center indicates the exact physical position of the marker.

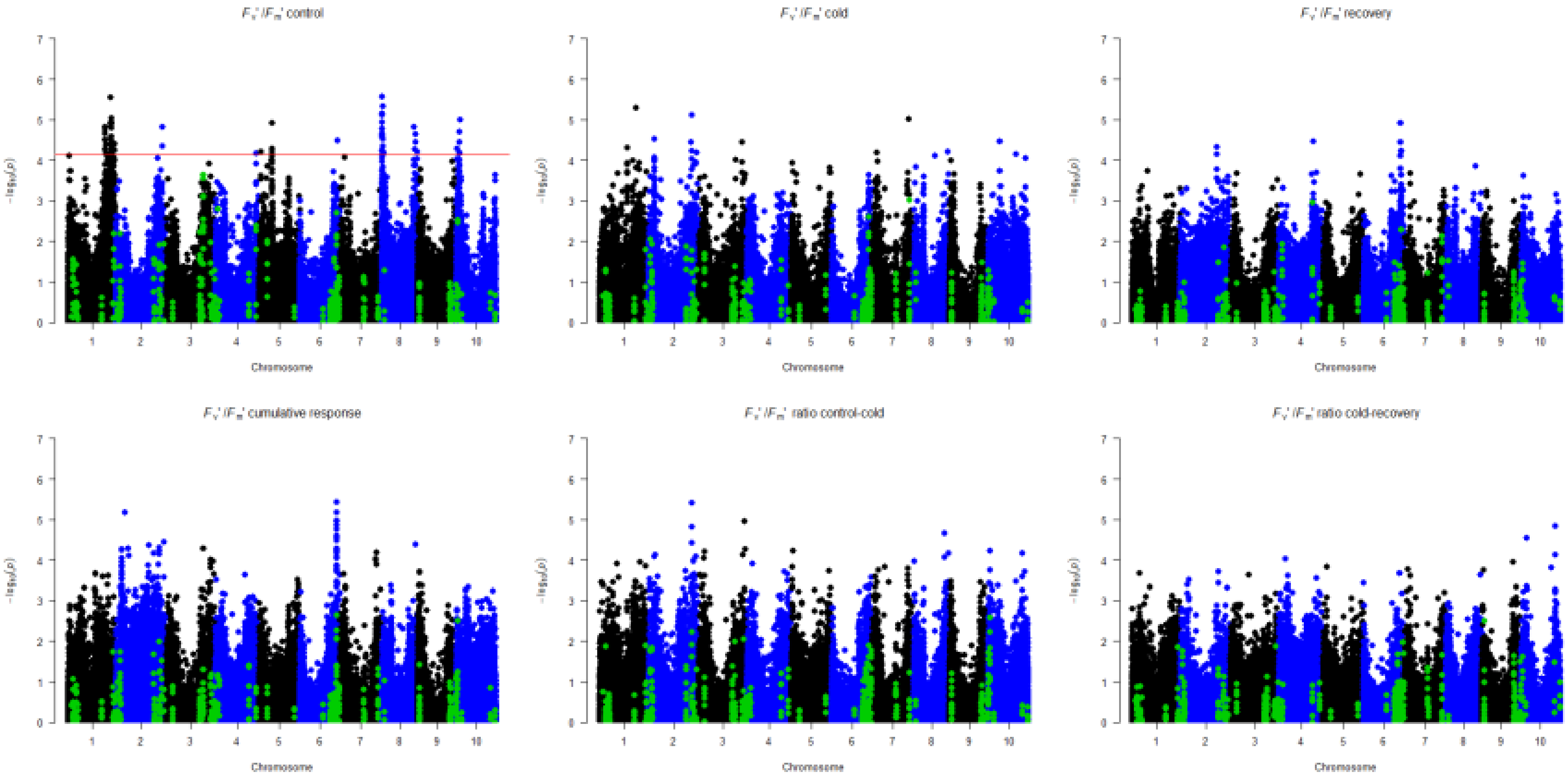

Supplementary Figure S10. Genome-wide association study results for $F_v´/F_m´$ in three temperature treatments and in response area using 304 diverse sorghum accessions. Horizontal red line indicates significance threshold. Green dots indicate the physical position of *a priori* candidate genes. Each single-nucleotide polymorphism is represented by a dot, whose center indicates the exact physical position of the marker.

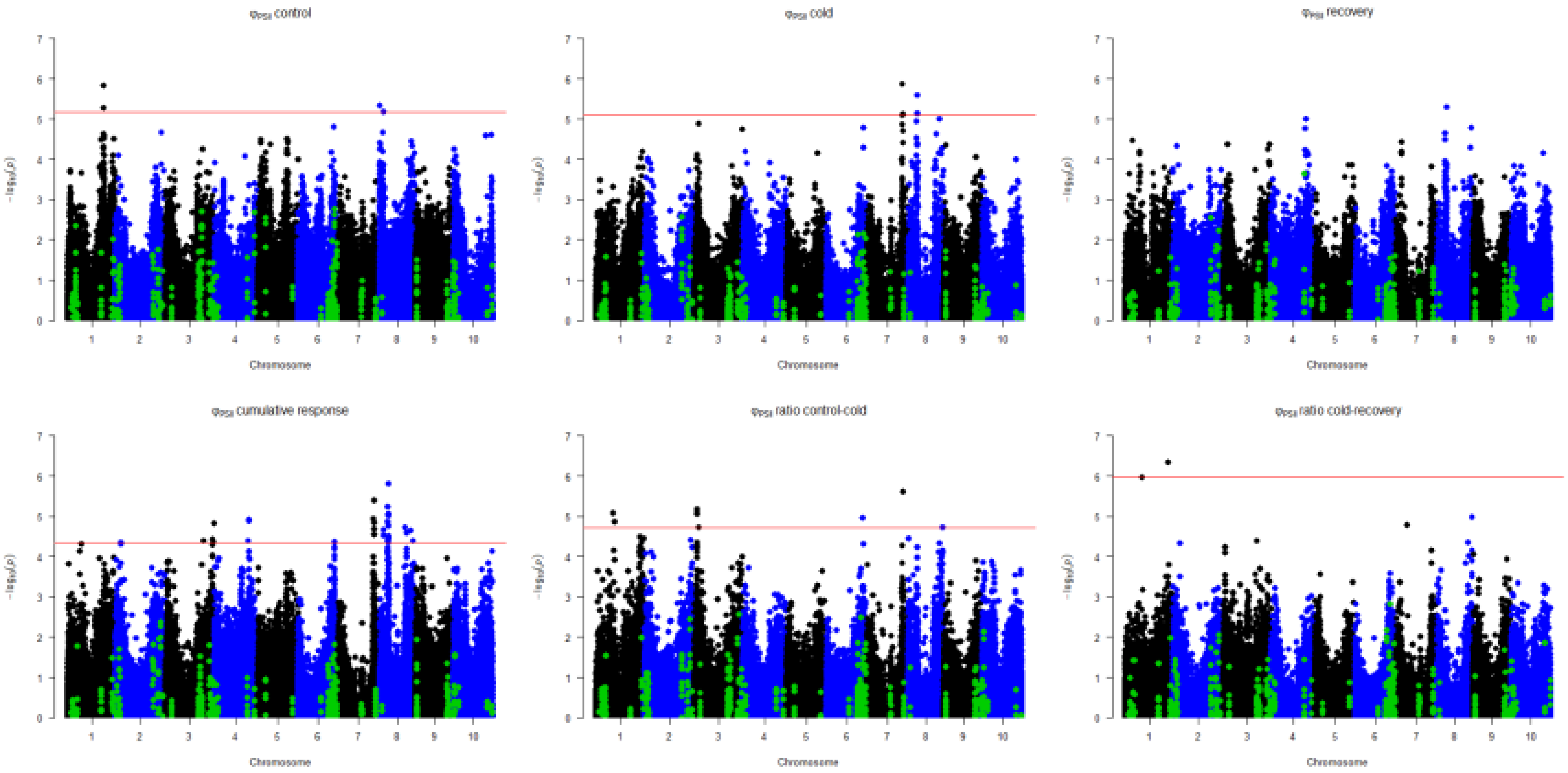

Supplementary Figure S11. Genome-wide association study results for Φ_{PSII} in three temperature treatments and in response area using 304 diverse sorghum accessions. Horizontal red line indicates significance threshold. Green dots indicate the physical position of *a priori* candidate genes. Each single-nucleotide polymorphism is represented by a dot, whose center indicates the exact physical position of the marker.

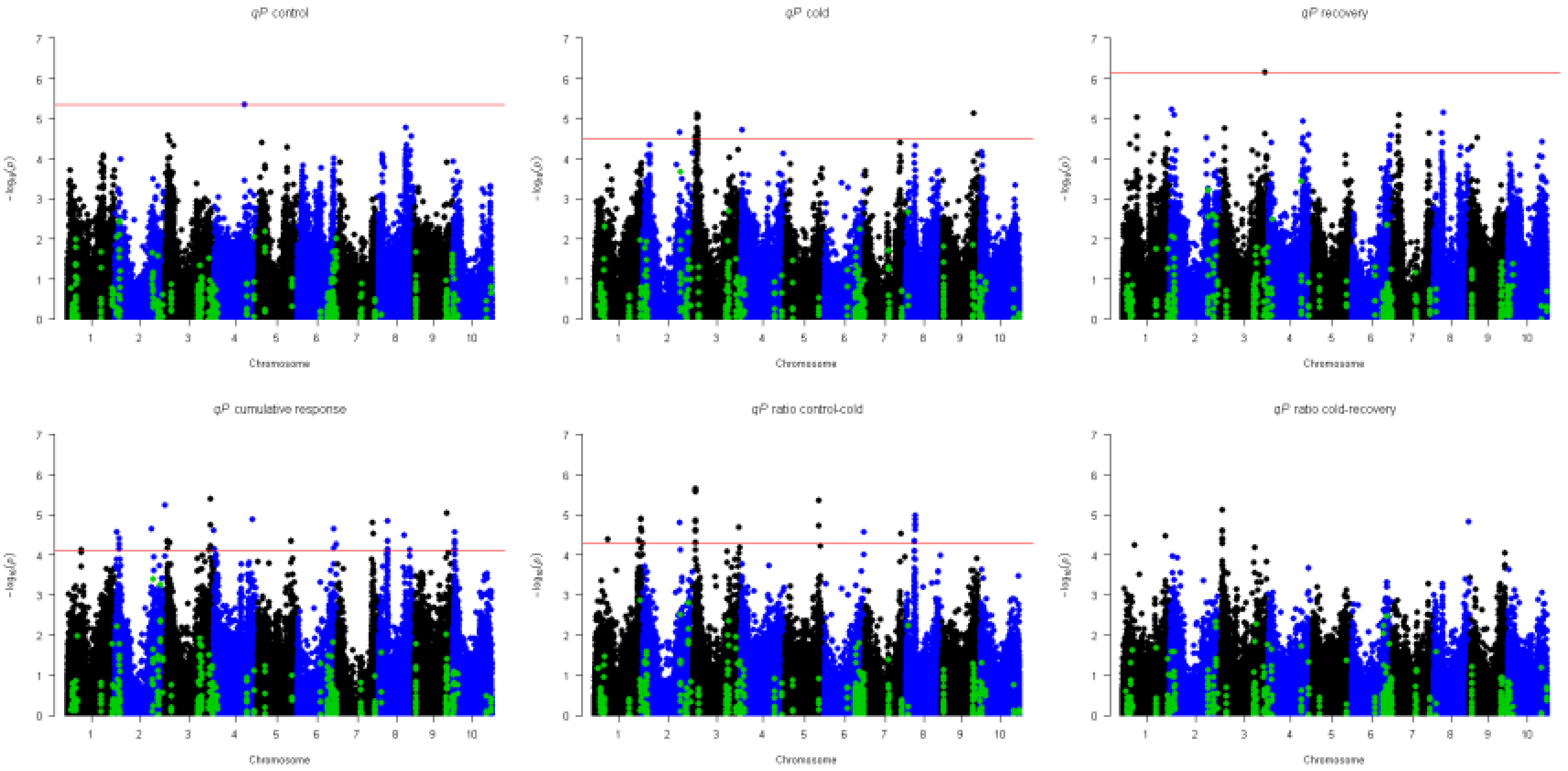

Supplementary Figure S12. Genome-wide association study results for *qP* in three temperature treatments and in response area using 304 diverse sorghum accessions. Horizontal red line indicates significance threshold. Green dots indicate the physical position of *a priori* candidate genes. Each single-nucleotide polymorphism is represented by a dot, whose center indicates the exact physical position of the marker.

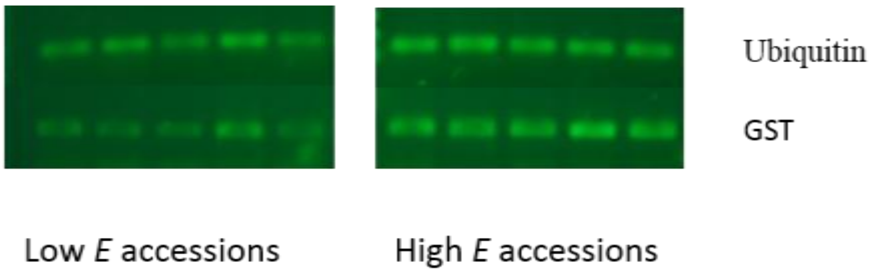

Supplementary Figure S13. RT-PCT expression levels for genes *UBIQUITIN* and *GLUTATHIONE S-TRANSFERASE* (*GST*) (*Sb08g007310*), in accessions presenting low and high *E* in the cold temperature treatment. Lanes from left to right correspond to accessions: for low *E* PI534115, PI597964, PI656071, PI656048, and PI595714; and for high *E*: PI656103, PI656036, PI534137, PI656013, and PI533754.
