# Supplementary material for: Genetic architecture of photosynthesis in Sorghum bicolor under non-stress and cold stress conditions
Source: J Exp Bot. 2017 Aug 18;68(16):4545–57. doi: 10.1093/jxb/erx276 (PMC5853419; doi:10.1093/jxb/erx276)
Supplement: supplementary_tables_S1_S7 [file erx276_suppl_supplementary_tables_s1_s7.pdf]

Genetic architecture of photosynthesis in *Sorghum bicolor* under non-stress and cold stress conditions

Diego Ortiz, Jieyun Hu, Maria G. Salas Fernandez

Supplemental Table S1. Phenotypic correlations between photosynthesis and chlorophyll fluorescence traits based on BLUPs in control period.

	Correlation (*r*)						
Traits	*A*	*E*	g_s	F_v/F_m	F_v'/F_m'	Φ_{PSII}	*qP*
A	-						
E	0.81***	-					
g_s	0.84***	0.95***	-				
F_v/F_m	0.32***	0.25***	0.23***	-			
F_v'/F_m'	0.69***	0.58***	0.59***	0.32***	-		
Φ_{PSII}	0.91***	0.71***	0.75***	0.28***	0.75***	-	
qP	0.74***	0.53***	0.57***	0.13*	0.25***	0.82***	-

A= photosynthesis (μmol CO_2 m^{-2} s^{-1}) ; *E*= transpiration rate (mmol H_20 m^{-2} s^{-1}), g_s= stomatal conductance (mol H_20 m^{-2} s^{-1}), F_v/F_m = maximum quantum yield of PSII, Φ_{PSII}=effective quantum yield of PSII; F_v'/F_m'=efficiency of energy captured by open PSII reaction centers and *qP*=photo-chemical quenching or fraction of PSII reaction centers that are open; *Significant at $P < 0.05$;** Significant at $P < 0.01$;*** Significant at $P < 0.001$

Supplemental Table S2. Phenotypic correlations between photosynthetic and chlorophyll fluorescence traits based on BLUPs in cold period

	Correlation (*r*)						
Traits	*A*	*E*	g_s	F_v/F_m	F_v'/F_m'	Φ_{PSII}	*qP*
A	-						
E	0.72***	-					
g_s	0.68***	0.96***	-				
F_v/F_m	0.28***	0.22***	0.24***	-			
F_v'/F_m'	0.43***	0.51***	0.52***	0.52***	-		
Φ_{PSII}	0.96***	0.7***	0.65***	0.28***	0.47***	-	
qP	0.77***	0.48***	0.42***	-0.07	-0.13*	0.78***	-

A= photosynthesis (μmol CO_2 m^{-2} s^{-1}) ; *E*= transpiration rate (mmol H_20 m^{-2} s^{-1}), g_s= stomatal conductance (mol H_20 m^{-2} s^{-1}), F_v/F_m = maximum quantum yield of PSII, Φ_{PSII}=effective quantum yield of PSII; F_v'/F_m'=efficiency of energy captured by open PSII reaction centers and *qP*=photo-chemical quenching or fraction of PSII reaction centers that are open; *Significant at $P < 0.05$;** Significant at $P < 0.01$;*** Significant at $P < 0.001$

Supplemental Table S3. Phenotypic correlations between photosynthetic and chlorophyll fluorescence traits based on BLUPs in recovery period.

Traits	Correlation (*r*)						
	A	*E*	g_s	F_v/F_m	F_v'/F_m'	Φ_{PSII}	*qP*
A	-						
E	0.94***	-					
g_s	0.96***	0.98***	-				
F_v/F_m	0.49***	0.46***	0.47***	-			
F_v'/F_m'	0.82***	0.78***	0.81***	0.6***	-		
Φ_{PSII}	0.97***	0.9***	0.93***	0.5***	0.83***	-	
qP	0.87***	0.79***	0.8***	0.32***	0.56***	0.91***	-

A= photosynthesis (μmol CO_2 m^{-2} s^{-1}) ; *E*= transpiration rate (mmol H_20 m^{-2} s^{-1}), g_s= stomatal conductance (mol H_20 m^{-2} s^{-1}), F_v/F_m = maximum quantum yield of PSII, Φ_{PSII}=effective quantum yield of PSII; F_v'/F_m'=efficiency of energy captured by open PSII reaction centers and *qP*=photo-chemical quenching or fraction of PSII reaction centers that are open; *Significant at $P < 0.05$;** Significant at $P < 0.01$;*** Significant at $P < 0.001$

Supplemental Table S4. Phenotypic correlations between photosynthetic and chlorophyll fluorescence traits evaluated as cumulative response, using BLUPs.

	Correlation (*r*)						
Traits	A	E	g_s	F_v/F_m	F_v'/F_m'	Φ_{PSII}	qP
A	-						
E	0.93***	-					
g_s	0.9***	0.97***	-				
F_v/F_m	0.5***	0.47***	0.49***	-			
F_v'/F_m'	0.66***	0.68***	0.69***	0.68***	-		
Φ_{PSII}	0.97***	0.89***	0.86***	0.5***	0.67***	-	
qP	0.81***	0.7***	0.65***	0.18**	0.19**	0.85***	-

A= photosynthesis (μmol CO_2 m^{-2} s^{-1}) ; E= transpiration rate (mmol H_20 m^{-2} s^{-1}), g_s= stomatal conductance (mol H_20 m^{-2} s^{-1}), F_v/F_m = maximum quantum yield of PSII, Φ_{PSII}=effective quantum yield of PSII; F_v'/F_m'=efficiency of energy captured by open PSII reaction centers and qP=photo-chemical quenching or fraction of PSII reaction centers that are open; *Significant at P < 0.05;** Significant at P < 0.01;*** Significant at P < 0.001

Supplemental Table S5. Phenotypic correlations between photosynthetic and chlorophyll fluorescence traits evaluated as ratio control-cold, using BLUPs.

	Correlation (*r*)						
Traits	*A*	*E*	g_s	F_v/F_m	F_v'/F_m'	Φ_{PSII}	*qP*
A	-						
E	0.78***	-					
g_s	0.76***	0.94***	-				
F_v/F_m	0.24***	0.19***	0.2***	-			
F_v'/F_m'	0.61***	0.55***	0.55***	0.39***	-		
Φ_{PSII}	0.96***	0.75***	0.71***	0.21***	0.64***	-	
qP	0.8***	0.58***	0.53***	-0.02	0.11	0.82***	-

A= photosynthesis (μmol CO_2 m^{-2} s^{-1}) ; *E*= transpiration rate (mmol H_20 m^{-2} s^{-1}), g_s= stomatal conductance (mol H_20 m^{-2} s^{-1}), F_v/F_m = maximum quantum yield of PSII, Φ_{PSII}=effective quantum yield of PSII; F_v'/F_m'=efficiency of energy captured by open PSII reaction centers and *qP*=photo-chemical quenching or fraction of PSII reaction centers that are open; *Significant at $P < 0.05$;** Significant at $P < 0.01$;*** Significant at $P < 0.001$

Supplemental Table S6. Phenotypic correlations between photosynthetic and chlorophyll fluorescence traits evaluated ratio cold-recovery, using BLUPs.

	Correlation (r)						
Traits	A	E	g_s	F_v/F_m	F_v'/F_m'	Φ_{PSII}	qP
A	-						
E	0.77***	-					
g_s	0.77***	0.95***	-				
F_v/F_m	0.28***	0.23***	0.22***	-			
F_v'/F_m'	0.63***	0.57***	0.59***	0.36***	-		
Φ_{PSII}	0.81***	0.54***	0.53***	0.14*	0.6***	-	
qP	0.6***	0.31***	0.28***	-0.02	0.01	0.77***	-

A= photosynthesis (μmol CO_2 m^{-2} s^{-1}) ; E= transpiration rate (mmol H_20 m^{-2} s^{-1}), g_s= stomatal conductance (mol H_20 m^{-2} s^{-1}), F_v/F_m = maximum quantum yield of PSII, Φ_{PSII}=effective quantum yield of PSII; F_v'/F_m'=efficiency of energy captured by open PSII reaction centers and qP=photo-chemical quenching or fraction of PSII reaction centers that are open; *Significant at $P < 0.05$;** Significant at $P < 0.01$;*** Significant at $P < 0.001$

Supplemental Table S7. Analysis of variance of photosynthesis and chlorophyll fluorescence traits in in control, cold and recovery periods

	A control				*A* cold				*A* recovery			
Fixed effects	Num DF	Den DF	F Value	Pr > F	Num DF	Den DF	F Value	Pr > F	Num DF	Den DF	F Value	Pr > F
Day	2	659	11	<.0001	1	662	923	<.0001	1	647	217	<.0001
machine	2	661	9	<.0001	2	141	10	<.0001	2	136	7	0.001
Tleaf					1	731	20	<.0001				
Random effects	Variance	LR p-value	% variation		Variance	LR p-value	% variation		Variance	LR p-value	% variation	
set	1.59	0.074	13		1.35	0.009	15		8.46	0.015	11	
Rep(set)	0.97	<.0001	8		0.29	0.001	3		1.93	0.011	2	
Geno	3.14	<.0001	26		2.49	<.0001	28		22.66	<.0001	29	

	E control				*E* cold				*E* recovery			
Fixed effects	Num DF	Den DF	F Value	Pr > F	Num DF	Den DF	F Value	Pr > F	Num DF	Den DF	F Value	Pr > F
Day	2	654	15	<.0001	1	660	710	<.0001	1	664	111	<.0001
machine	2	829	29	<.0001	2	161	4	0.024	2	93	2	0.207
Random effects	Variance	LR p-value	% variation		Variance	LR p-value	% variation		Variance	LR p-value	% variation	
set	0.02519	0.317	9		0.01016	0.046	10		0.09662	0.014	9	
Rep(set)	0.04328	<.0001	16		0.00422	<.0001	4		0.01926	0.028	2	
Geno	0.05179	<.0001	19		0.02107	<.0001	21		0.21630	<.0001	21	

	g_s control				g_s cold				g_s recovery			
Fixed effects	Num DF	Den DF	F Value	Pr > F	Num DF	Den DF	F Value	Pr > F	Num DF	Den DF	F Value	Pr > F
Day	2	645	37	<.0001	1	660	789	<.0001	1	658	202	<.0001
machine	2	915	16	<.0001	2	114	3	0.038	2	114	3	0.049
Random effects	Variance	LR p-value	% variation		Variance	LR p-value	% variation		Variance	LR p-value	% variation	
set	0.00000	1.000	0		0.00009	0.043	8		0.00039	0.009	13	
Rep(set)	0.00015	<.0001	17		0.00003	0.007	3		0.00007	0.014	2	
Geno	0.00024	<.0001	26		0.00028	<.0001	23		0.00074	<.0001	24	

	F_v/F_m control				F_v/F_m cold				F_v/F_m recovery			
Fixed effects	Num DF	Den DF	F Value	Pr > F	Num DF	Den DF	F Value	Pr > F	Num DF	Den DF	F Value	Pr > F
Day	2	658	21	<.0001	1	640	286	<.0001	1	628	171	<.0001
Random effects	Variance	LR p-value	% variation		Variance	LR p-value	% variation		Variance	LR p-value	% variation	
set	0.00003	0.001	24		0.00007	0.083	4		4.59E-06	1.000	0.1	
Rep(set)	2.91E-06	0.001	2		0.00002	0.254	1		0.00004	0.030	1	
Geno	0.00003	<.0001	26		0.00025	<.0001	13		0.00022	0.005	6	

	F_v'/F_m' control				F_v'/F_m' cold				F_v'/F_m' recovery			
Fixed effects	Num DF	Den DF	F Value	Pr > F	Num DF	Den DF	F Value	Pr > F	Num DF	Den DF	F Value	Pr > F
Day	2	662	39	<.0001	1	659	449	<.0001	1	663	219	<.0001
machine	2	406	32	<.0001	2	128	1	0.375	2	137	0	0.641
Random effects	Variance	LR p-value	% variation		Variance	LR p-value	% variation		Variance	LR p-value	% variation	
set	0.00002	0.439	3		0.00011	0.294	3		0.00080	0.021	12	
Rep(set)	0.00005	<.0001	6		0.00013	0.004	4		0.00020	0.011	3	
Geno	0.00018	<.0001	24		0.00075	<.0001	22		0.00123	<.0001	18	

	Φ_{PSII} control				Φ_{PSII} cold				Φ_{PSII} recovery			
Fixed effects	Num DF	Den DF	F Value	Pr > F	Num DF	Den DF	F Value	Pr > F	Num DF	Den DF	F Value	Pr > F
Day	2	658	41	<.0001	1	649	1104	<.0001	1	663	180	<.0001
machine	2	691	107	<.0001	2	87	22	<.0001	2	159	3	0.080
Random effects	Variance	LR p-value	% variation		Variance	LR p-value	% variation		Variance	LR p-value	% variation	
set	0.00023	0.024	21		0.00015	0.006	13		0.00049	0.069	7	
Rep(set)	0.00009	<.0001	8		0.00002	0.017	2		0.00021	0.004	3	
Geno	0.00025	<.0001	22		0.00036	<.0001	31		0.00179	<.0001	25	

	qP control				*qP* cold				*qP* recovery			
Fixed effects	Num DF	Den DF	F Value	Pr > F	Num DF	Den DF	F Value	Pr > F	Num DF	Den DF	F Value	Pr > F
Day	2	659	14	<.0001	1	655	303	<.0001	1	661	62	<.0001
machine	2	906	90	<.0001	2	115	16	<.0001	2	169	4	0.013
Random effects	Variance	LR p-value	% variation		Variance	LR p-value	% variation		Variance	LR p-value	% variation	
set	0.00057	0.027	29		0.00129	0.002	20		0.00080	0.121	6	
Rep(set)	0.00024	<.0000	12		0.00017	0.003	3		0.00053	0.001	4	
Geno	0.00032	<.0001	16		0.00178	<.0001	27		0.00322	<.0001	24	

A= photosynthesis (μmol CO_2 m^{-2} s^{-1}) ; *E*= transpiration rate (mmol H_20 m^{-2} s^{-1}), g_s= stomatal conductance (mol H_20 m^{-2} s^{-1}), F_v/F_m = maximum quantum yield of PSII, Φ_{PSII}=effective quantum yield of PSII; F_v'/F_m'=efficiency of energy captured by open PSII reaction centers and *qP*=photo-chemical quenching or fraction of PSII reaction centers that are open; LR= Likelihood ratio; Tleaf= temperature of the leaf; Num DF= numerator of degrees of freedom; Den DF= Denominator of degrees of freedom
